# Responsible agriculture must adapt to the wetland character of mid‐latitude peatlands

**DOI:** 10.1111/gcb.16152

**Published:** 2022-03-17

**Authors:** Benjamin W. J. Freeman, Chris D. Evans, Samuel Musarika, Ross Morrison, Thomas R. Newman, Susan E. Page, Giles F. S. Wiggs, Nicholle G. A. Bell, David Styles, Yuan Wen, David R. Chadwick, Davey L. Jones

**Affiliations:** ^1^ 1506 School of Natural Sciences Bangor University Bangor Gwynedd UK; ^2^ UK Centre for Ecology and Hydrology Bangor Gwynedd UK; ^3^ UK Centre for Ecology and Hydrology Wallingford Oxfordshire UK; ^4^ 4488 School of Geography, Geology and the Environment University of Leicester Leicester Leicestershire UK; ^5^ 6396 School of Geography and the Environment University of Oxford Oxford Oxfordshire UK; ^6^ School of Chemistry University of Edinburgh Edinburgh Midlothian UK; ^7^ Ryan Institute National University of Ireland Galway Galway Ireland; ^8^ 34752 College of Agronomy and Biotechnology China Agricultural University Beijing China; ^9^ SoilsWest Centre for Sustainable Farming Systems Food Futures Institute Murdoch University Murdoch Western Australia Australia

**Keywords:** carbon, climate change mitigation, greenhouse gases, hydrology, paludiculture, peatlands, soil loss, wetland agriculture

## Abstract

Drained, lowland agricultural peatlands are greenhouse gas (GHG) emission hotspots and a large but vulnerable store of irrecoverable carbon. They exhibit soil loss rates of ~2.0 cm yr^−1^ and are estimated to account for 32% of global cropland emissions while producing only 1.1% of crop kilocalories. Carbon dioxide emissions account for >80% of their terrestrial GHG emissions and are largely controlled by water table depth. Reducing drainage depths is, therefore, essential for responsible peatland management. Peatland restoration can substantially reduce emissions. However, this may conflict with societal needs to maintain productive use, to protect food security and livelihoods. Wetland agriculture strategies will, therefore, be required to adapt agriculture to the wetland character of peatlands, and balance GHG mitigation against productivity, where halting emissions is not immediately possible. Paludiculture may substantially reduce GHG emissions but will not always be viable in the current economic landscape. Reduced drainage intensity systems may deliver partial reductions in the rate of emissions, with smaller modifications to existing systems. These compromise systems may face fewer hurdles to adoption and minimize environmental harm until societal conditions favour strategies that can halt emissions. Wetland agriculture will face agronomic, socio‐economic and water management challenges, and careful implementation will be required. Diversity of values and priorities among stakeholders creates the potential for conflict. Successful implementation will require participatory research approaches and co‐creation of workable solutions. Policymakers, private sector funders and researchers have key roles to play but adoption risks would fall predominantly on land managers. Development of a robust wetland agriculture paradigm is essential to deliver resilient production systems and wider environmental benefits. The challenge of responsible use presents an opportunity to rethink peatland management and create thriving, innovative and green wetland landscapes for everyone's future benefit, while making a vital contribution to global climate change mitigation.

## Graphical abstract

Peatlands are a globally important but diminishing and irrecoverable carbon store. Hydrology is a dominant driver of peatland ecosystem function, and water table depth is a strong predictor of peatland greenhouse gas emissions. Responsible management requires agriculture to adapt to the wetland character of peatlands. Wetland agriculture strategies could increase the resilience of production systems, deliver a wider range of environmental benefits and protect these valuable ecosystems for everyone's future benefit, whilst making a vital contribution to global climate change mitigation efforts.

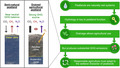

## 1 INTRODUCTION

The Agriculture, Forestry and Other Land Use (AFOLU) sector contributes ~24% of global greenhouse gas (GHG) emissions (Smith et al., 2014). Agricultural production will need to rise by ~60% to meet global food demands by 2050 (Alexandratos & Bruinsma, 2012), and mitigation measures will be required to limit associated increases in agricultural GHG emissions (Bennetzen et al., 2016). The challenge of balancing climate change mitigation and adaptation with achieving food security has been formally recognised by policymakers in the Paris Agreement (UNFCCC, 2015) and is particularly acute for agriculturally managed peatlands.

Global peatlands store >600 Gt of carbon (C) in an estimated area of 4.23 million km^2^ (Xu et al., 2018; Yu et al., 2010). This represents more C than was added to the atmosphere by total anthropogenic carbon dioxide (CO_2_) emissions between 1750 and 2011, stored on less than 3% of the global land area (IPCC, 2014). Many peatlands have been drained to enhance the delivery of economically valuable provisioning services (e.g. food and timber). Consequently, there are an estimated 500,000 km^2^ of heavily modified, diminishing peatlands globally, emitting 1.2–1.9 Gt CO_2_‐e yr^−1^ and contributing ~14% of AFOLU GHG emissions (Joosten, 2010; Leifeld & Menichetti, 2018). Halting these emissions will be integral to achieving climate stabilization (Günther et al., 2020). This review focuses on mid‐latitude (non‐tropical and non‐polar; S1) peatlands, which contain ~75% of peatland C and account for ~22% of modified peatland GHG emissions (Leifeld & Menichetti, 2018).

Agriculture is responsible for ~50% of the peatland conversion that has occurred in mid‐latitude areas, with the greatest impacts seen on relatively accessible lowland fens and raised bogs (Joosten & Clarke, 2002). Forestry and peat extraction account for much of the remainder. Drainage has facilitated the application of conventional agricultural systems, which originated in dryland regions, to naturally wet lowland peatlands, producing highly productive agroecosystems. However, it is estimated that peatland agriculture accounts for 32% of global cropland GHG emissions, despite producing only 1.1% of total crop kilocalories (Carlson et al., 2017). Peatland C stocks are irrecoverable, as C depleted by drainage cannot be replenished over human‐relevant timescales (Noon et al., 2021). Therefore, conventional agriculture on peatlands is neither economically nor environmentally sustainable (Wijedasa et al., 2016). Given the cultural and economic significance of peatland agriculture to many of the regions where it occurs, high levels of private land ownership and the absence of mechanisms to reflect the high external costs of peatland GHG emissions, full restoration to pre‐drainage condition and function is unlikely to be immediately viable in all cases. Full rewetting and complete halting of peatland GHG emissions should remain a long‐term goal. However, it is imperative that responsible peatland management strategies are developed, which adapt productive agricultural management to the wetland character of peatlands and slow peat loss/emission rates; minimizing harm until societal conditions favour halting emissions (Clarke & Rieley, 2019). This review aims to (i) critically assess the impacts resulting from drainage‐based agriculture on mid‐latitude lowland peatlands, (ii) evaluate the potential for wetland agriculture systems to enable responsible peatland management and (iii) highlight key challenges, which must be addressed to inform research priorities, and support land managers and policymakers in this vital undertaking.

## 2 AGRICULTURAL DRAINAGE IMPACTS

Peat soils (histosols) are those with more than 50% organic material in the top 80 cm, or with shallower organic deposits (C content >12–18%) resting directly on bedrock and not influenced by permafrost (USDA Soil Survey Staff, 1999). Peatlands form when impeded drainage produces waterlogged conditions and the rate of organic matter accumulation exceeds the rate of decomposition. As a result, undisturbed mid‐latitude peatlands act as a net C sink (~ −0.32 t C ha^−1^ yr^−1^) and have a net climate cooling impact on millennial timescales; on a 100‐year global warming potential (GWP) basis, the balance of CO_2_, methane (CH_4_) and nitrous oxide (N_2_O) results in a small net GHG source (Frolking et al., 2011).

**FIGURE 1:**
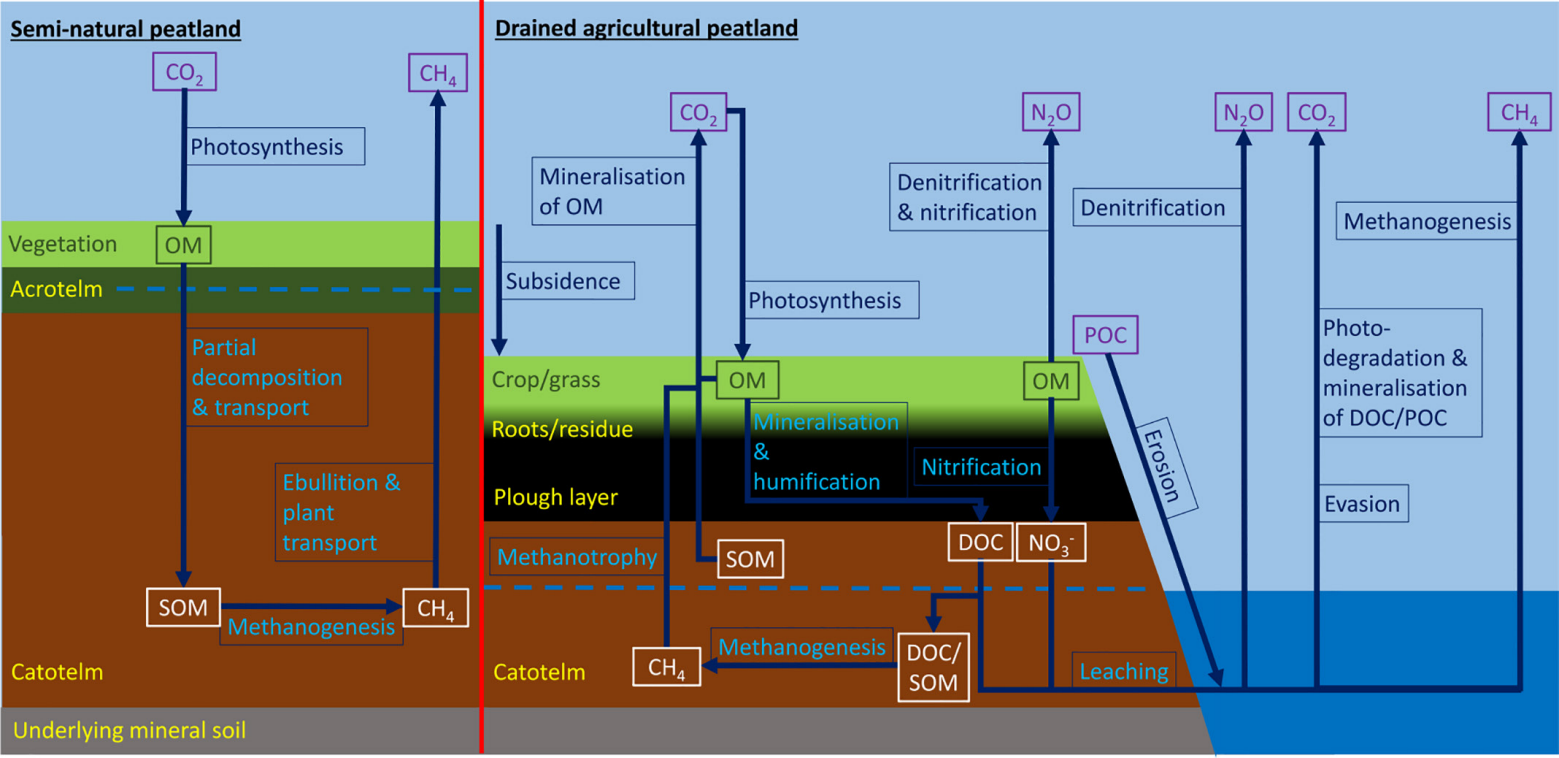
Impacts of drainage for agriculture on fundamental peatland processes. Blue dashed lines indicate the average water table depth (WTD) with fluctuation around this level assumed. Semi‐natural peatlands are approximately carbon neutral but can be slight net sinks or sources of greenhouse gas (GHG) emissions depending on methane emissions. Drained peatlands are strong sources of GHG emissions from both fields and ditches. The acrotelm is the partially aerated upper layer of semi‐natural peatlands, while the catotelm is the submerged, anaerobic, lower peat layer. Fluctuations in the WTD produce a dynamic mesotelm layer between these, which has been omitted for clarity. Additions of fertiliser and livestock excreta increase labile carbon (C) and nitrogen (N) stocks in agricultural peatlands, exacerbating changes in C and N cycling. The fate of dissolved organic material leached from semi‐natural peatlands to streams and rivers is similar to that shown for drained peatlands and is omitted from the diagram in the interest of space. CH_4_, methane; CO_2_, carbon dioxide; DOC, dissolved organic carbon; N_2_O, nitrous oxide; OM, organic matter; POC, particulate organic carbon; SOM, soil organic matter

Conversion to the deeply drained agricultural landscapes, characteristic of today's mid‐latitude lowland peatlands began with gravity drainage and accelerated sequentially with the harnessing of wind‐power (11th Century AD), the advent of steam power and the centrifugal water pump (1800s), and most recently modern diesel engines and electric pumps (Sly, 2010). Drainage has a range of impacts on peatland function (Figure 1). The most visible impact is subsidence of the land surface, which enhances flood risk and causes costly damage to infrastructure (Page et al., 2020). Subsidence results from physical shrinkage of organic matter, compaction of the peat pore spaces, microbial mineralisation of soil organic matter (SOM) and increased vulnerability to erosion. Measured subsidence rates from the literature are relatively consistent across mid‐latitude drained agricultural peatlands, with a median rate of 2.0 cm yr^−1^ (Quartiles = 1.3–2.7, *n* = 48; S2).

Mineralisation of SOM has been estimated to account for 28–64% of subsidence in a temperate climate (Leifeld et al., 2011). In the early years following drainage, primary subsidence is rapid, dominated by shrinkage and compaction, and results in large volumetric losses. However, it is subsequent, more gradual, secondary subsidence, with larger contributions from mineralisation, which results in the depletion of SOM/C stocks, until eventually, the loss of peat depth and C content proceeds to such an extent that the soil is no longer classifiable as peat (Hutchinson, 1980).

Drainage results in increased oxygenation and thus soil redox potential, which facilitates aerobic respiration of soil microbes and increases CO_2_ emissions. This emission is substantial and dominates the overall GHG balance of the ecosystem (Table 1). Using available emission factors (EFs) for temperate agricultural peatlands, the estimated contribution of soil CO_2_ emissions to the terrestrial GHG balance is 82.9% for cropland, 89.7% for intensive grassland and 83.9% for extensive grassland (S3). It will, therefore, be necessary to reduce terrestrial CO_2_ emissions if substantial mitigation of GHG emissions is to be achieved.

**TABLE 1:** Tier 1 emission factors for mid‐latitude peatlands under agricultural management

Emission factor	Land use	Climate zone	Nutrient status	Drainage depth	Value	LCI	UCI	*N*
CO_2_ (t CO_2_‐C ha^−1^ yr^−1^)	Cropland	Boreo‐temperate			7.9	6.5	9.4	39
	Grassland	Boreal			5.7	2.9	8.6	8
		Temperate	Low		5.3	3.7	6.9	39
			High	Deep	6.1	5	7.3	7
				Shallow	3.6	1.8	5.4	13
Field CH_4_ (kg CH_4_ ha^−1^ yr^−1^)	Cropland	Boreo‐temperate			0	−2.8	2.8	38
	Grassland	Boreal			1.4	−1.6	4.5	12
		Temperate	Low		1.8	0.72	2.9	9
			High	Deep	16	2.4	29	44
				Shallow	39	−2.9	81	16
N_2_O (kg N_2_O‐N ha^−1^ yr^−1^)	Cropland	Boreo‐temperate			13	8.2	18	36
	Grassland	Boreal			9.5	4.6	14	16
		Temperate	Low		4.3	1.9	6.8	7
			High	Deep	8.2	4.9	11	47
				Shallow	1.6	0.56	2.7	13
Ditch CH_4_ (kg CH_4_ ha^−1^ yr^−1^)	Agriculture	Boreo‐temperate		Deep	1165	335	1995	6
	Grassland	Boreo‐temperate		Shallow	527	285	769	5
DOC (t C ha^−1^ yr^−1^)	Agriculture	Boreal			0.12	0.07	0.19	^a^
	Agriculture	Temperate			0.31	0.19	0.46	^a^

Abbreviations: LCI/UCI, lower/upper 95% confidence intervals; *n*, number of studies included in deriving estimate.

^a^ Single value not available for composite metric. Values collated from Drösler et al. (2014).

Mineralisation of SOM also releases nitrogen (N), resulting in high mineral‐N supplies (e.g. 250–571 kg N ha^−1^ yr^−1^ for cropland; Rochette et al., 2010). Where these exceed crop N demands and provide substrate for microbial metabolism, substantial N_2_O emissions can occur (Table 1; Poyda et al., 2016). Emissions of N_2_O can represent an important component of the terrestrial GHG balance for cropland (17%; S3) and intensively managed grassland (9.2%; S3).

In drained peatlands, CH_4_ oxidation potentials are high throughout the soil profile (Jerman et al., 2017), which results in near‐zero terrestrial CH_4_ emissions from croplands (Table 1). Grassland soil CH_4_ emissions are also generally low but can be high during periods of inundation and rapid anaerobic decomposition of flood‐intolerant grassland plant species (Tiemeyer et al., 2016). These conditions are mostly observed on shallow‐drained, extensive grassland sites, where CH_4_ emissions can constitute an important portion of the terrestrial GHG balance (9.8%; S3).

Methanogenesis is a major catabolic process in anoxic environments and can proceed rapidly in drainage ditches bordering fields. Observed ditch CH_4_ fluxes are highly variable and currently poorly understood but can be substantial (Table 1) and may make an important contribution to overall site GHG budgets (Peacock et al., 2021).

Ditches also represent an export pathway for dissolved and particulate organic C (DOC and POC, respectively). In watercourses, this C is vulnerable to photodegradation and can be transformed by microbial activity, resulting in indirect CO_2_ emissions, which may occur far from the peatland (e.g. in rivers, lakes or coastal seas), while dissolved CO_2_ exported directly from the peat is generally released rapidly (Evans et al., 2016a). Indirect CO_2_ emissions from DOC and POC export are generally low relative to terrestrial emissions in drained systems (Table 1).

Secondary humification produces small, light particles, which are highly susceptible to wind erosion when exposed. The severity of wind erosion events on cultivated peatlands has long been known (Thompson, 1957), but measurements of their magnitude and dynamics are extremely rare. Cumming (2018) recorded sediment movements at the border of an arable UK peatland field and observed sediment fluxes of 0.87–4.88 t C ha^−1^ yr^−1^. Peak fluxes coincided with periods of bare soil and high wind speed, suggesting losses may be much lower from permanent grassland. The ultimate fate of eroded material remains unclear, so it is not currently possible to estimate the contribution of aeolian losses to depletion of C stocks or indirect GHG emissions.

Anthropogenic drainage reduces peatland moisture content, increasing their flammability and the depth of peat available for combustion (Turetsky et al., 2011). Fire impacts can, therefore, be severe, with a typical uncontrolled fire estimated to emit 122 t CO_2_‐C ha^−1^ (Drösler et al., 2014). Controlled burning still takes place on some mid‐latitude peatlands but the practice is rare, largely due to the air pollution impacts, demonstrated by recent fires in Russia and the United Kingdom (Chubarova et al., 2009; Graham et al., 2020). Accidental fire risks remain on abandoned sites where drainage may be poorly managed but these can be reduced by rewetting (Sirin et al., 2020).

Drainage‐induced mineralisation of SOM is the dominant factor driving long‐term subsidence, C stock depletion and GHG emissions in agriculturally managed peatlands. Consequently, suppressing rates of SOM mineralisation and CO_2_ emissions is the primary pro‐environmental objective required for responsible peatland management. N_2_O and CH_4_ emissions can also make important contributions to GHG balances and must be considered, along with provisions to mitigate erosion losses and fire risk.

## 3 WATER TABLE CONTROL OF EMISSIONS

Average annual peatland subsidence rates are linearly related to the average annual water table depth (WTD), increasing by an estimated 0.2 cm yr^−1^ for every 0.1 m of additional drainage in non‐tropical peatlands (Evans et al., 2019). This result is derived from long‐term subsidence data, including sites with long drainage histories. Very long‐term studies show decreasing subsidence rates over time (Hutchinson, 1980; Stephens et al., 1984) and rapid primary subsidence immediately following drainage was not included in this analysis. Average annual WTD strongly influences the volume of aerated organic matter and thus microbial activity, making it a very convenient indicator of peatland function. It is a simple measure, which cannot capture site‐specific differences in moisture content, oxygen concentration and C density in the unsaturated zone. Variables such as average summer WTD (Weideveld et al., 2021) and hydrograph skewness (Tiemeyer et al., 2016) may offer more nuance. However, average annual WTD has been widely used in the literature to date and data are more often available. We, therefore, adopt this measure of WTD unless otherwise stated.

**FIGURE 2:**
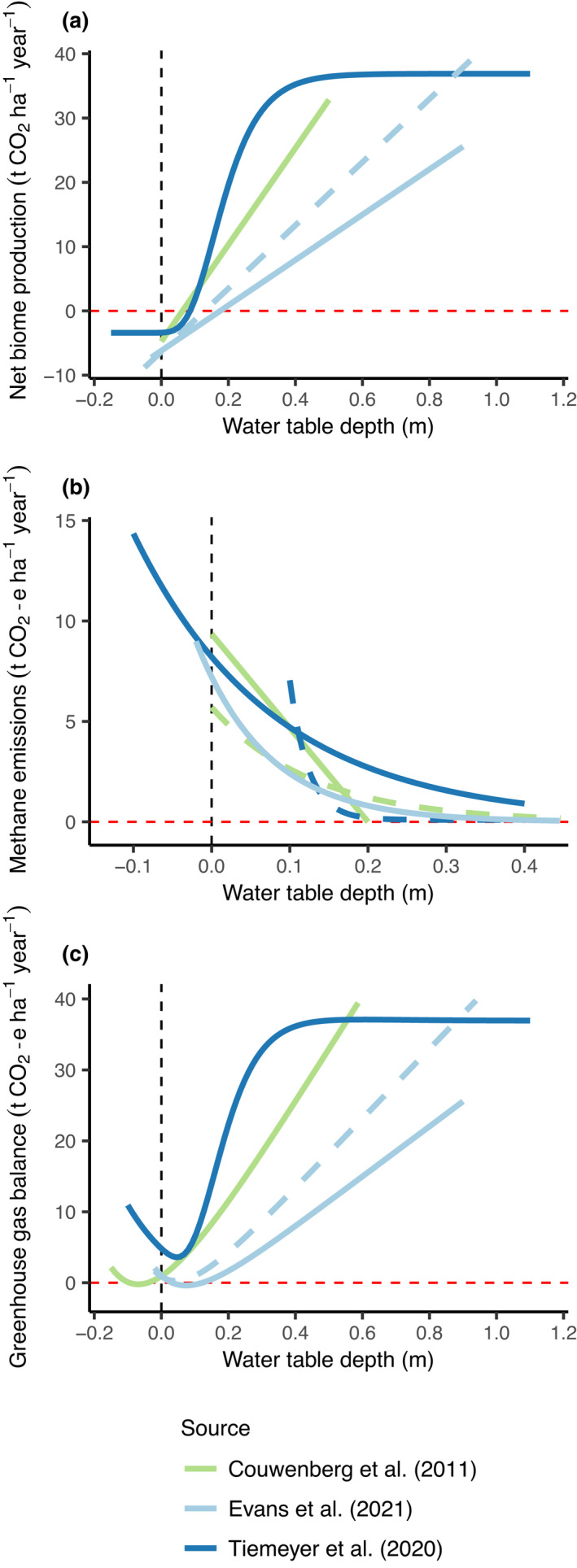
Relationships between peatland water table depth (WTD) and carbon‐derived greenhouse gas emissions. (a) Net biome production (NBP; sum of ecosystem respiration, gross primary productivity and carbon import/export). Dashed and solid light blue lines represent UK and global relationships, respectively, in Evans et al. (2021). (b) Terrestrial methane emissions (CH_4_; excluding ditch emissions and converted to CO_2_ equivalent using a 100‐year global warming potential of 28). Dashed and solid dark blue lines represent relationships for agricultural and rewetted sites, respectively, in Tiemeyer et al. (2020). Solid and dashed green lines indicate the published relationship from Couwenberg et al. (2011) and an exponential function fitted to a digitized subset of these data (see S4 for detailed description). (c) Terrestrial GHG balance of CO_2_ (NBP) and CH_4_. Functions for Tiemeyer et al. (2020) and Couwenberg et al. (2011) produced using rewetted site and exponential CH_4_ functions, respectively. Dashed and solid light blue lines as for (a). Vertical dashed black lines indicate the peat surface (WTD = 0 m). More positive WTD values indicate deeper drainage and negative values indicate inundation. Horizontal red dashed lines indicate emission values of zero

Several data syntheses have shown a positive relationship between CO_2_ emissions and peatland WTD (Figure 2a; Couwenberg et al., 2011; Evans et al., 2021; Tiemeyer et al., 2020). The slope and shape of the fitted relationships vary between these studies. However, there is strong agreement that (i) CO_2_ emissions are high on drained agricultural peatlands (mean predicted value = 24.7 ± 5.9 t CO_2_ ha^−1^ yr^−1^ when WTD = 0.5 m) and (ii) surface‐level WTDs on semi‐natural sites result in net CO_2_ uptake (mean predicted value = −5.2 ± 0.7 t CO_2_ ha^−1^ yr^−1^ when WTD = 0 m). Experimental manipulations of WTD remove potentially confounding differences between land uses and still generally support this trend for WTDs ≤ 0.7 m (e.g. Karki et al., 2014; Regina et al., 2015). Overall, the clear implication is that WTDs nearer the surface are linked to lower rates of SOM mineralisation and CO_2_ emissions in peatlands. This analysis also indicates that no new peatland drainage should occur if overall peatland GHG emissions are to be reduced.

Terrestrial CH_4_ emissions from peatlands are consistently very low at WTDs deeper than 0.25 m (Figure 2b). However, a sharp increase is observed as WTDs are reduced beyond this level. There is agreement across several data syntheses regarding this exponential response (Couwenberg et al., 2011; Evans et al., 2021; Tiemeyer et al., 2020). In balancing CO_2_ against CH_4_, C‐derived GHG emissions appear to be minimised when the WTD is close to the peat surface (Figure 2c). The mean optimal WTD indicated for C‐derived‐GHG mitigation is 0.04 ± 0.03 m based on a 100‐year GWP of 28 for CH_4_ (mean prediction of the four functions shown in Figure 2c plus the Tiemeyer et al. (2020) GHG balance using agricultural site CH_4_ emissions not shown in Figure 2c; Myhre et al., 2013). This is slightly deeper (0.08 ± 0.02 m) when estimated using a 100‐year sustained GWP of 45 for CH_4_ (Neubauer & Megonigal, 2015). Further inundation increases CH_4_ emissions, offsetting CO_2_ reductions and indicating that flooded peatlands would be GHG sources.

**FIGURE 3:**
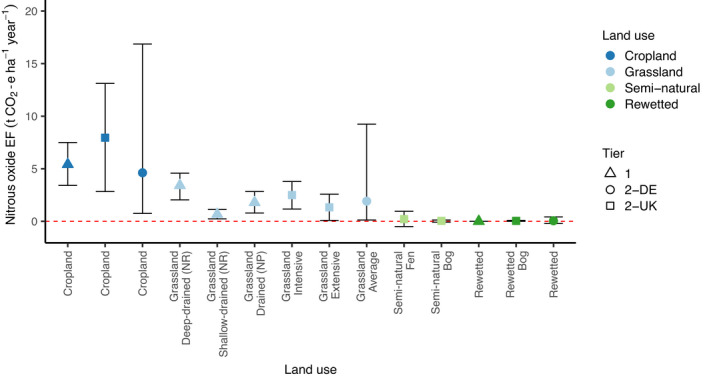
Nitrous oxide emission factors for selected land‐use categories. Error bars indicate 95% confidence intervals (CIs). The horizontal red dashed line indicates zero emissions and is included to highlight that the CIs for cropland and grassland sites exclude zero, while the CIs for semi‐natural and rewetted sites include zero. N_2_O was converted to CO_2_ equivalent using a 100‐year global warming potential of 265 (Myhre et al., 2013) to aid comparison with carbon‐derived greenhouse gas emissions. Land‐use categories are presented in approximate order of decreasing water table depth (WTD), with deeper drained agricultural sites on the left and near‐surface WTDs on the right. NR, nutrient‐rich and NP, nutrient‐poor. Tier 1 (Default) emissions factors (EFs) were sourced from Drösler et al. (2014), Tier 2 (Germany; DE) EFs from Tiemeyer et al. (2020) and Tier 2 (United Kingdom; UK) EFs from Evans et al. (2017)

Deeply drained agricultural sites exhibit higher N_2_O emissions than near‐natural sites and so the overall pattern is of increasing N_2_O emissions with deeper peatland WTDs (Figure 3; Leppelt et al., 2014). However, as for CO_2_, management practices (e.g. N inputs and vegetation) potentially confound this relationship and the situation appears more complex within land‐use categories. Tiemeyer et al. (2016) found that the extent of WTD fluctuations and topsoil N stocks best predicted N_2_O emissions from German peat grasslands, while rainfall (Taghizadeh‐Toosi et al., 2019) and irrigation (Rochette et al., 2010) have also been observed to stimulate N_2_O emissions from agricultural peatlands. Hot moments, driven by the onset of winter flooding, irrigation and fertilisation, accounted for 45% of annual N_2_O emissions from a peat cropland in California (Anthony & Silver, 2021). These studies highlight the risk of large, denitrification‐driven N_2_O pulses when drained soils are subject to acute wetting events and WTD fluctuations. This is especially the case following prolonged periods of drainage, when mineralisation and nitrification of SOM, along with anthropogenic N inputs, lead to nitrate (NO_3_^−^) accumulation, providing plentiful substrate for denitrification upon subsequent wetting (Taghizadeh‐Toosi et al., 2019). N_2_O emissions are likely to be low from consistently fully saturated peat, due to inhibition of nitrification and complete denitrification to N_2_ (Taft et al., 2018).

The evidence implies that minimising GHG emission rates from agricultural peatlands will require maintenance of shallower and more stable WTDs, alongside reduced soil mineral‐N concentrations. While near‐surface WTDs would be optimal for GHG mitigation, there is some indication that smaller reductions in WTD, deeper in the peat profile, may partially mitigate GHG emissions (Evans et al., 2021). However, such partial changes could only be expected to slow peat loss rates, with mineralisation continuing in the aerated layer and leading to eventual loss of the peat. Excessive inundation has the potential to induce substantial CH_4_ emissions, and may also constrain primary productivity, peat formation and associated CO_2_ uptake. Because of the strong control that WTD exerts over C‐derived GHG emission rates (the majority of the GHG budget), WTD management clearly represents the most efficacious tool available to slow the rate of peat loss. Responsible WTD management is, therefore, essential for responsible peatland management. Development of wetland agriculture systems will be necessary, to adapt agricultural production to the wetland character of peatlands, and ensure continued delivery of provisioning services alongside improved environmental outcomes.

## 4 WETLAND AGRICULTURE SYSTEMS

### 4.1 Paludiculture systems

Responsible peatland management requires a balance to be struck between GHG mitigation, food security and economic productivity. Rewetting and restoring peatlands to semi‐natural conditions can achieve substantial reductions in the rate of GHG emissions (Nugent et al., 2019) and will represent an important component of national and international climate strategies. However, it greatly reduces delivery of provisioning services from peatlands, which will not be desirable in all cases. Paludiculture is a form of wetland agriculture, which pairs near‐surface WTDs with production systems compatible with the resulting wet conditions (Wichtmann et al., 2016; Table 2). It, therefore, represents a compromise approach, weighted strongly towards GHG mitigation but without complete loss of the provisioning services prioritised under the currently dominant paradigm of drainage‐based, conventional agriculture.

**TABLE 2:** Overview of relationships between conventional systems and wetland agriculture systems

Category	Sub‐category	Summer WTD	Winter WTD	Area under modified WTD	Land use on modified WTD area	Land use on unmodified WTD area
Conventional agriculture	Conventional agriculture	Deep drained (BAU)	Deep drained (BAU)	None	N/a	Conventional agriculture
Wetland agriculture	Paludiculture	Near‐surface	Near‐surface	Whole site	Paludiculture	N/a
	Reduced drainage depth	Intermediate	Intermediate	Whole site	Adapted agriculture	N/a
	Reduced drainage duration	Deep drained (BAU)	Near‐surface	Whole site	Adapted agriculture	N/a
	Reduced drainage area	Near‐surface	Near‐surface	Part of site only	Paludiculture or restoration	Conventional agriculture

Note

There is a subdivision between paludiculture and reduced drainage intensity approaches in the extent of modification. Reductions in overall drainage intensity are separated into those decreasing the depth, duration or area of drainage. However, in practice some combination of these may also be used. These are broad characterizations intended to highlight differences. Water table depths (WTDs) and management practices on specific sites may be less clear‐cut.

Abbreviation: BAU, business as usual.

Several paludiculture systems have been identified that can substantially reduce soil loss rates. Cultivation of biomass species for the production of bioenergy and biofuels on rewetted peatlands in northern Europe has shown potential to produce near‐neutral onsite CO_2_ balances even after biomass removal (Günther et al., 2015; Kandel et al., 2017). Bioenergy from Reed Canary Grass (*Phalaris arundinacea*) has been estimated to produce ~40% lower GHG emissions per unit of energy generated than coal combustion and to result in a negative overall GHG balance (Järveoja et al., 2013; Shurpali et al., 2010). The wet conditions favoured by some paludiculture crops can induce CH_4_ emissions, reducing the favourability of their GHG balance on a 100‐year GWP basis and requiring careful management (e.g. Günther et al., 2015). However, the suitability of using 100‐year GWP to assess the warming equivalence of short‐lived pollutants is questionable (Cain et al., 2019). The relatively short atmospheric lifespan of CH_4_, means that adoption of these systems may still be favoured as it would result in a beneficial climatic effect over time, due to avoided emissions of more atmospherically persistent CO_2_ (Günther et al., 2020).

Peat moss (*Sphagnum* spp.) production sites can be net CO_2_ sinks during cultivation and may be essentially C neutral after accounting for biomass harvest (Beyer & Höper, 2015; Günther et al., 2017). The GHG balance over the full life cycle may be negative due to avoided emissions if horticultural peat extraction were replaced by *Sphagnum* cultivation. However, commercially viable *Sphagnum* cultivation faces practical challenges. Farmed *Sphagnum* is liable to be more expensive than extracted peat and it is currently unclear whether it can fully replicate peat's properties as a growing medium (Mulholland et al., 2020). Biomass and *Sphagnum* cultivation systems are both subject to high initial investments and harvesting costs, as specialist machinery is required to manage wet peatlands (Mulholland et al., 2020; Wichmann, 2017).

Food production options on wet peatlands are limited for mid‐latitude regions. Wet meadows and pasture can provide fodder and grazing for livestock production, and extensively managed grassland sites can have GHG balances close to semi‐natural peatlands (Beetz et al., 2013). However, they have low biomass production rates and, therefore, only support low stocking rates, so profitability is generally low in the current economic landscape. Given enteric CH_4_ emissions from ruminant livestock and generally high GHG emissions from meat production, there is a risk of substituting GHG emission sources if additional livestock production results from this change. The UK Committee on Climate Change recently factored reduced meat consumption into its projections of future land‐use sector emissions (Stark et al., 2020), suggesting that changes which increase livestock production may not always be commensurate with wider societal goals. Few conventional crops are tolerant of flooded conditions. Paddy rice (*Oryza sativa*) production is well established, suitable for fen peatlands and can reduce GHG emissions by ~75% relative to drained cropland (Knox et al., 2015). Its viable geographic range is climate dependent and water demands can be substantial, which currently constrains its applicability within the mid‐latitudes.

Paludiculture adoption in the mid‐latitudes would likely reduce food production on the peatland area. This is an important consideration, given global food security goals. Adoption also risks displacing food production elsewhere, entailing higher agrochemical or transportation burdens, and counterbalancing direct benefits. However, due to the finite lifespan of drained agricultural peatlands, their high productivity is only temporary. Sustainable intensification of agriculture on mineral soils, alongside demand‐side measures and waste reductions will be essential to meet global food requirements (Springmann et al., 2018). Where possible, proactive peatland restoration and transition to mineral soil production would allow protection of peatland C stocks and retention of regulating/supporting ecosystem services of substantial value (Wichmann et al., 2016).

The limited available evidence suggests that paludiculture would generally result in lower agricultural profitability than conventional agriculture within the current economic landscape and so it will not be immediately viable in all circumstances (Mulholland et al., 2020; Wichmann, 2017). However, paludiculture offers an important strategy for mitigation of GHG emissions and the protection of peatland C stocks, without the complete loss of agricultural profitability associated with full restoration. With a favourable economic environment including efficient C markets for emissions reductions/sequestration and an adequate C price, such systems could play an important role in responsible peatland management (de Jong, 2020). The possibility also exists that peatlands could be managed as active C/GHG sinks (‘C farms’) based on paludiculture‐type approaches without biomass harvest to maximise net‐uptake (Element Energy and UKCEH, 2021). Development of functioning paludiculture demonstration systems could support identification of economic potential and development of appropriate GHG EFs at field and product level (Tanneberger et al., 2020). Delivering these research outcomes is vital to support decision‐makers in both policy and industry to invest in the development of commercially viable paludiculture options.

### 4.2 Reduced drainage intensity systems

Where socio‐economic circumstances are currently incompatible with restoration or paludiculture adoption, alternative wetland agriculture systems will be required. These would need to impose smaller reductions in the delivery of provisioning services but would require a compromise on their GHG mitigation potential. Reduced drainage intensity systems may deliver such a compromise and would involve some combination of reducing the drainage depth, the duration of drainage and the area drained (Table 2). The aim would be to maximise the proportion of the peat layer at a site that is saturated over the course of the year and thus produce partial, but potentially significant, reductions in rates of subsidence and GHG emissions, while minimising impacts on agricultural production.

The evidence suggests that bringing the average WTD closer to the peat surface might incrementally reduce C‐derived GHG emissions up to a WTD of ~0.05 m. Several mesocosm studies have confirmed that WTD reductions from 0.5 m to 0.3 m can significantly reduce CO_2_ emissions from agricultural peat soils, without significantly increasing CH_4_ emissions (Matysek et al., 2019; Musarika et al., 2017; Wen et al., 2020). Of these studies, only Wen et al. (2020) measured N_2_O emissions, observing a 41% reduction. However, this study did not add N, which can exacerbate N_2_O emissions in wet peatland systems (Kandel et al., 2019; Wen et al., 2021). The effect of WTD change on N_2_O emissions from active agricultural peatlands remains poorly quantified and represents an important weakness in our understanding.

Current agricultural practices rely predominately on crops that originated in dryland regions and are poorly suited to wet conditions. Varying, yield effects were observed in the aforementioned mesocosm studies for celery (−19%; *Apium* graveolens; Matysek et al., 2019), lettuce (−37%; *Lactuca* sativa; Wen et al., 2020) and radish (+33%; *Raphanus raphanistrum*; Musarika et al., 2017). Where yield decreases occur, they are likely to be economically significant and in the absence of external costs being borne by producers, either compensation (e.g. payments for avoided emissions) or income from the sale of C credits (for emission reductions) may be required to offset losses (Buschmann et al., 2020). Robust studies of the impact of WTD management on crop yield and quality are required to support decision‐makers with implementation of reduced drainage depth systems on cropland. Alternative crops, which can tolerate wetter soil conditions may be necessary to adapt agriculture to reduced drainage depths (e.g. Cranberries and Blueberries—*Vaccinium* spp.; Abel, 2016).

Land‐use change from cropland to grassland is an option to reduce the WTD to at most 0.5 m, while limiting reductions in agricultural profitability. Intensive grasslands do require drainage but shallower WTDs are possible under grass than most conventional crops. WTDs around 0.5 m support grassland biomass production (Campbell et al., 2015), while producing bearing capacities generally suitable for vehicle access (Schothorst, 1982). Consequently, such systems are already widespread but as noted above, GHG emissions resulting from any additional livestock production may offset reductions in peat‐derived GHG emissions, potentially limiting the net benefits of such land‐use change.

Long‐term drainage ultimately causes sufficient loss (or ‘wastage’) of peat depth and SOM content that the soil no longer meets the definition of a histosol. In areas of Northern Europe that have been drained for centuries, such soils are widespread, and in some regions may comprise the majority of the agricultural ‘peatland’ area. Robust EFs for wasted peat soils represent an important knowledge gap, with studies indicating that CO_2_ emissions decrease with declining SOM content (Taft et al., 2017), are similar to those from deeper peat soils (Tiemeyer et al., 2016) or are higher from lower SOM peat soils (Leiber‐Sauheitl et al., 2014). This uncertainty extends to the scale, and nature of, mitigation measures required.

Reducing WTDs on agriculturally active wasted peatlands would be extremely challenging. Near‐surface WTDs would be needed to saturate a substantial proportion of the remaining peat layer and wastage often reveals topographically uneven subsoils. One option for reducing drainage intensity on these sites may be to reduce drainage duration, and thus the time for which the peat layer is aerated. Approximately 23–41% of net CO_2_ emissions occur during the winter (October–March; Evans et al., 2016b), when farm activity is reduced, potentially providing an opportunity to reduce drainage depths. Wen et al. (2020) observed 33% lower GHG emissions from mesocosms during the winter at a WTD of 0.3 m compared with 0.5 m. Arable production is possible on seasonally flooded peat in California (winter WTD up to −0.3 m), although viability may rely on adequate evapotranspiration rates, which will vary with climate (Anthony & Silver, 2021). Shallower winter WTDs may restrict vehicle access and interfere with field preparation. They could also lead to shifts in grassland plant community composition, but this requires prolonged wet conditions and may be avoidable with appropriate management (Toogood & Joyce, 2009).

Where reductions in neither drainage depth nor intensity are deemed possible (e.g. wasted peat cropland in cool climates), reducing the area under drainage may be the only option to achieve substantial GHG emission reductions. This could be achieved by placing some portion of a site under rewetted conditions (e.g. paludiculture/restoration), while continuing conventional production on the remainder.

Reduced drainage intensity systems are likely to offer less GHG mitigation, but lead to smaller reductions in agricultural profitability, compared with paludiculture in the current regulatory landscape. However, we currently lack robust field and experimental evidence of the effects of reduced drainage depth and duration on GHG emissions and crop yield/quality. Field trials at plot and field scale will be necessary to evaluate the benefits of these approaches, to allow the identification and resolution of any management issues, and to enable optimal management of the trade‐off between GHG mitigation and economic productivity.

Development of reduced drainage intensity systems is an important step in producing robust wetland agriculture options for responsible management of mid‐latitude peatlands. These options do not equate to truly sustainable management, because decomposition will continue in the remaining aerated peat layer. Therefore, they do not argue against peatland restoration or paludiculture adoption where these are suitable. However, reduced drainage intensity systems are closer to the status quo than restoration/paludiculture adoption, and may, therefore, meet with fewer practical, socio‐economic and political barriers. If solutions to challenges can be found, they may, therefore, make a significant contribution to the overall mitigation of agricultural peatland GHG emission rates and provide a transitional option where circumstances preclude immediate restoration or paludiculture adoption.

### 4.3 Drainage and water resource management

**FIGURE 4:**
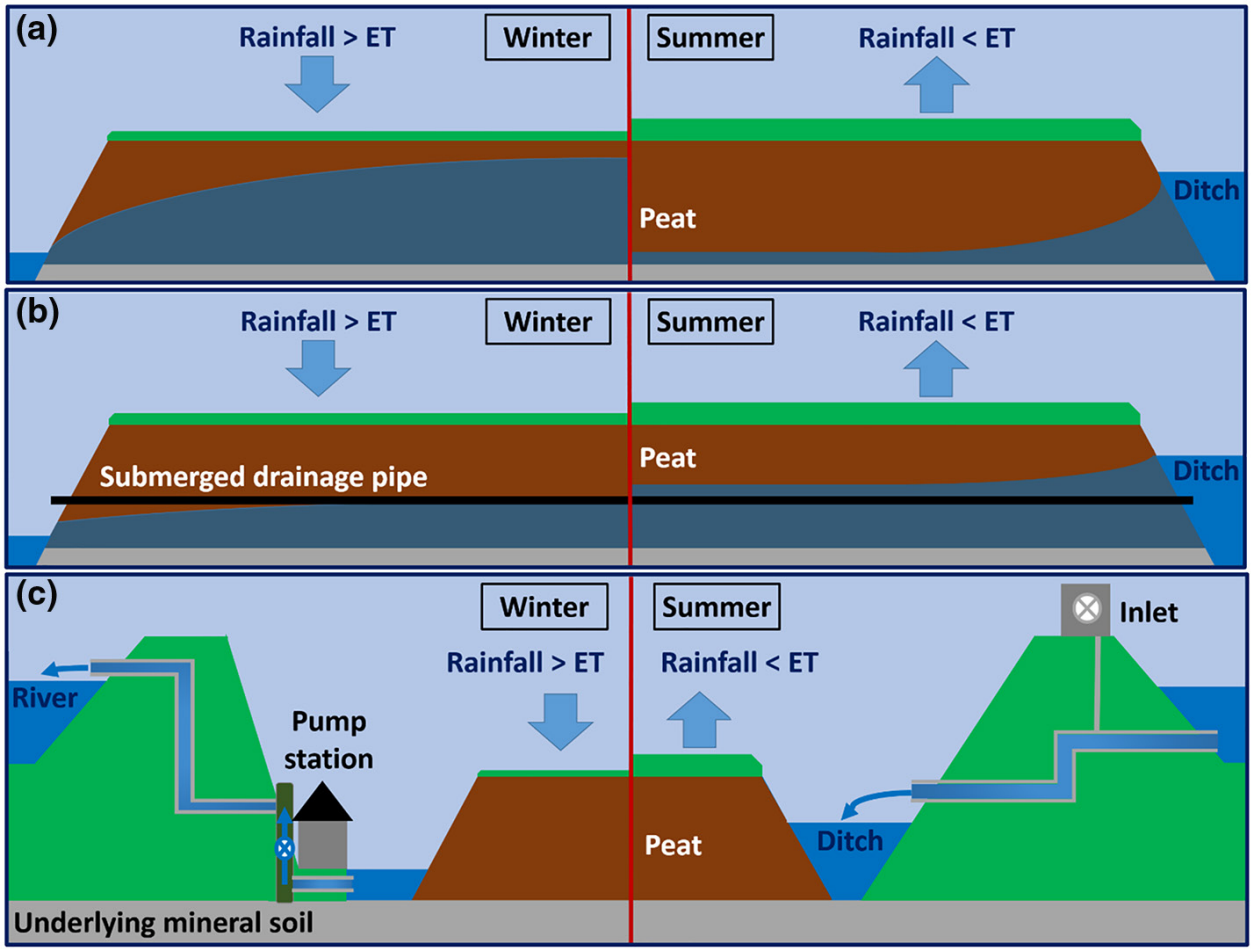
Seasonal water management in agricultural lowland peatlands. (a) Field water table conditions with drainage ditches alone, (b) Theoretical field water table conditions with submerged drains, (c) Drainage management on agricultural peatlands subject to extensive subsidence. In winter, water is pumped from ditches up to rivers, to drain the fields and limit flood risk. In summer, water is allowed to flow down from rivers to ditches to aid irrigation of the crop/sward. Sub‐images (a) and (b) developed from Hoving et al. (2015)

The drainage infrastructure and technology required for management of wetland agriculture systems has not yet been fully developed, and is of varying quality in different regions. The management of drainage and water resources is likely to pose challenges for wetland agriculture adoption at both field and regional scales. Reduced drainage intensity systems will require close regulation of WTDs within fields to minimise agricultural risks. Traditional drainage ditch networks were designed for removal of excess water in winter and are usually unsuitable for precise WTD control. Submerged drainage systems involve the installation of drainage pipes within the peat layer to improve drainage in winter and limit WTD drawdown in summer (Weideveld et al., 2021; Figure 4a and b). This can provide a more stable WTD over the course of the year, facilitating both sub‐irrigation of crops and winter vehicle access. However, they may perform better in aiding drainage than in producing infiltration of water into the field (Hoving et al., 2008). Their performance can be enhanced by manipulating ditch water levels relative to the drain depth (dynamic WTD management; Hoving et al., 2013, 2015) and by using pumps to adjust water levels in wells attached to submerged drainage pipes (Jansen et al., 2017). However, pumping will incur energy costs and indirect GHG emissions.

Submerged drainage systems almost certainly offer a valuable tool for improvement of field‐scale WTD control. It has been suggested that they can also reduce CO_2_ emissions by as much as 50% (van den Akker & Hendriks, 2017). However, this is based on an assumption of a linear relationship between subsidence rates and CO_2_ emissions and not direct GHG measurements (Couwenberg, 2018). A recent study suggests that submerged drainage alone does not lead to reductions in GHG emissions (Weideveld et al., 2021). We know of no studies that have measured GHG emissions while both (i) installing submerged drains and (ii) attempting to reduce the average annual WTD by simultaneously raising ditch levels relative to controls. Robust measurements of GHG emissions (including N_2_O) under such experimental conditions will be essential to understanding the GHG mitigation potential of wetland agriculture systems.

Many mid‐latitude agricultural peatland areas experience substantial seasonal variation in water availability. Subsidence means that many lowland peatlands lie below the level of local river channels and/or the sea, requiring major watercourses to be embanked to prevent river flooding. When regional precipitation exceeds the sum of evapotranspiration and available water storage capacity (e.g. in winter), surface flooding of fields will occur unless excess water is discharged by pumping (Figure 4c). Drainage pumping would continue to be necessary under paludiculture (Mulholland et al., 2020) and reductions in free storage capacity may also necessitate more rapid pumping to keep pace with incident rainfall and control flood risk. The rate of discharge from fields themselves may also present a challenge for reduced drainage depth strategies. Drainage and infrastructure improvements at both field and regional scale would almost certainly be required for wetland agriculture adoption. A better understanding of the hydrology of these systems will be needed to assess the scale of the resulting risks and constraints.

During the summer, evapotranspiration exceeds rainfall and water supply to meet crop/sward demands becomes the dominant challenge on agricultural peatlands. Water can be released from embanked watercourses to supplement agricultural requirements (Figure 4c). However, high demand and relatively low supply can create short‐term conditions of water stress even in regions where annual rainfall is abundant. These challenges would be likely to continue under wetland agriculture and additional water supply may be necessary (Querner et al., 2012). Reservoir construction may help to supplement the summer water supply by holding winter rainfall until it is needed and smoothing out seasonal trends in availability. However, the scale of infrastructure that would be required is unclear and may be substantial. Areas set aside for paludiculture, may form a dispersed network of emergency reservoirs; they could be managed for near‐surface WTDs normally, with allowances for drawdown to supplement crop irrigation under drought conditions. These areas could also perform a flood regulation role by holding excess water during winter.

Inevitably, different rates of adoption of wetland agriculture systems would risk conflict between land managers (Buschmann et al., 2020). In practice, hydrological isolation of sites will be challenging, because water management in agricultural peatlands typically occurs at the large (i.e. multiple farm) scale. Bunds and impermeable membranes have been used to reduce lateral water movement onto/off wet sites within agricultural peatland landscapes. However, these are rarely fully effective, and WTDs on adjacent sites are likely to influence each other. Power imbalances may exist due to proximity to water inputs or drainage pumps, although this might be manageable to some extent using bypass channels or sluice gates (Ferré et al., 2019). Managing the resulting patchwork of systems with varying WTDs and requirements will require land manager cooperation, coordination through administrative/regulatory bodies at larger scales and state support for the landscape‐scale infrastructure required. Regulators themselves will face challenges where conflict exists between various aspects of their role, suggesting that coherent policy will be essential to facilitate successful implementation.

Although the changes in drainage and water resource management required by wetland agriculture are substantial, they are currently poorly studied and there is a clear need to establish their efficacy and practicality. Our understanding of agricultural peatland hydrology is currently limited at both site and regional scales, but this is essential to understanding GHG mitigation potential in what are ultimately wetland systems. Addressing these limitations will be vital to evaluate the practical viability of establishing wetland agriculture systems, understand additional management challenges that may result from climate change and design appropriate land‐use policies.

### 4.4 *Socio*‐*economic considerations*

Agricultural peatlands are anthropogenic landscapes, providing both human habitation and livelihoods. Consequently, adoption of responsible management strategies cannot be considered in isolation from socio‐economic and political challenges (Table 3). Stakeholders experience a range of different pressures and exhibit differing preferences, resulting in value plurality and conflicting interests (Buschmann et al., 2020; Rawlins & Morris, 2010). Stakeholder analysis in the United Kingdom shows wide agreement on the important role of hydrological management on peatlands but a divergence on the importance of use (e.g. agriculture) and non‐use (e.g. pro‐environmental) values (Rawlins & Morris, 2010). This, along with the challenges of using peatlands without depleting them, has resulted in the current polarisation between heavily drained agricultural systems and wetland conservation/restoration sites. Wetland agriculture systems represent a compromise, balancing economic productivity and pro‐environmental outcomes, through a focus on improved hydrological management.

**TABLE 3:** Socio‐economic challenges facing wetland agriculture adoption on mid‐latitude peatlands

Challenge	Details
Opportunity costs	Agricultural use can be highly profitable.^1,2^ Where wetland agriculture is less profitable, this represents a loss if income is not replaced.^2,3^ Conversion costs borne by land managers cannot be invested in future productivity gains.^2^ Where changes are irreversible, perceived opportunity costs may be substantial.^4^
Uncertain time horizons	Remaining lifespans of agricultural peatlands vary between sites and are often uncertain.^1^ Uncertainty and poor visibility of soil loss rates on deep peat may affect perceptions of the urgency of response required.^1^
Uncertain costs of business as usual	Underlying mineral soils are variable and define the income generating potential after peat loss.^1^ Expectations of future yield enhancing technologies may mitigate concerns about transitioning to less productive underlying soils, reducing the perceived costs of continuing current practice.
Regional cost–benefit disparities	Spatial variation in productivity and C stocks will cause spatial variation in cost–benefit assessments around adoption. For example on highly productive systems with low remaining C stocks, the costs of offsetting production losses may outweigh the perceived benefits of adoption.^3^
Cultural identities	In many areas, agricultural use is long established and local communities have invested heavily in building rural economies.^1,4^ Pride in local culture and traditions may favour agricultural solutions and impede adoption of externally imposed novel solutions.^4^
Stakeholder networks	Highly connected networks, including both scientific expertise and local actors positioned to implement solutions, appear to enhance potential for adoption.^2^ Poorly connected networks are associated with low acceptance and potential for conflict.^2^
Stakeholder conflict	Local conflict may arise when adoption affects water levels on neighbouring land. Land‐use heterogeneity and high productivity can increase conflict potential.^3,5^ Larger‐scale conflict may arise over the importance of production and pro‐environmental ecosystem services.^6^
Economic pressures	Agricultural producers face pressures from retailers on both the quantity and timeframe of production.^1^ Producers unable to meet these demands under less productive/reliable systems may lose contracts or favourable terms, exacerbating profitability reductions.
Economic competition	Reduced production potential under wetland agriculture may diminish the comparative advantage of peatland production and expose producers to competition with mineral soil producers, leading to loss of market share and reducing profitability.^2^
Perceived locus of control and self‐efficacy	Perceptions of control and capacity are important precursors to pro‐environmental behaviours. Prescriptive, top‐down policy may reduce perceived control, while uncertainty around capacity for implementation may present an obstacle to adoption.^4^
Information availability/quality	Research is not always produced and communicated with the aim of providing useable information to end users.^6^ This effectively creates an information deficit, which may be exacerbated by low levels of trust towards researchers.^4^
Policy coherence	Implementation of mitigation measures can be impeded when national laws and land use/agricultural policies are not aligned with international/national climate policy.^3,6,7^
Incentivising mechanisms	There are currently few schemes formally incentivizing reduced WTDs on agricultural peatlands for climate mitigation and provision of public goods.^1^ Longer‐term schemes will be required to ensure persistence of WTD changes and provide security.^3^
Quantification of public goods	Lack of robust valuations for regulating, supporting and cultural ecosystem services delivered under mitigation measures leaves decision‐makers reliant on incomplete information.^6^
C market development	Peatland emissions are generally not eligible for compliance markets.^1,8^ Commodity C prices required to offset opportunity costs are often higher than current scheme prices.^1^
Indirect land‐use change impacts	Productivity declines associated with adoption may lead to production being exported to or intensified in other areas.^2^ Negative environmental effects elsewhere may, therefore, offset local benefits and generate resistance from relevant stakeholder groups.^1,2,5^

*Sources*: 1) Ferré et al. (2019), 2) Schaller et al. (2011), 3) Buschmann et al. (2020), 4) Reed et al. (2020), 5) Mulholland et al. (2020), 6) Rawlins and Morris (2010), 7) Regina et al. (2016) and 8) Bonn et al. (2014).

Abbreviations: C, carbon; WTD, water table depth.

Economic pressures will inevitably make short‐term costs of wetland agriculture adoption highly salient for land managers (Ferré et al., 2019). Contrastingly, the longer‐term economic costs of eventual peat loss are often unclear and quantification of the benefits of non‐production ecosystem services is notoriously challenging (Ferré et al., 2019; Rawlins & Morris, 2010). Furthermore, the wider benefits of these ecosystem services often occur at a societal level (e.g. flood regulation, water supply, landscape value and climate regulation) and are external to the land manager (Reed et al., 2014). The information deficit around peat losses/ecosystem service benefits is clearly an impediment to the development and adoption of wetland agriculture systems. In this context, hesitancy by decision‐makers is understandable, if not ideal given the urgency of response required.

The research community has an important role to play in overcoming this deficit. However, this will be impeded by low levels of trust towards researchers, who may not share land manager values (Reed et al., 2020). For example, substantial differences exist between solutions that researchers deem effective and those that land managers deem practical or economic (Taft, 2014). Strongly connected stakeholder networks, including both sources of scientific knowledge and local knowledge are associated with higher potential for adoption of wetland agriculture systems (Schaller et al., 2011). This suggests that researchers need to continue raising awareness about the unsustainability of drainage‐based, conventional agriculture on peatlands and the importance of WTD management. However, the successful development of a robust wetland agriculture paradigm will also require participatory research approaches, and the co‐creation of knowledge and workable solutions, alongside land managers.

The opportunity costs and initial capital investments required for wetland agriculture adoption mean that land managers will be reliant on changes in the economic landscape and an adequate C price to ensure economic viability (de Jong, 2020). Remuneration for delivery of public goods and pro‐environmental outcomes will be essential to support continued production where agricultural profitability is reduced. Private sector funding has the potential to play an important role, through corporate social responsibility and voluntary C markets. Land managers who can demonstrate verifiable reductions in (or cessation of) GHG emissions from peatlands may be well placed to secure private investment to support mitigation measures. However, successful harnessing of this capital pool will depend on the development of attractive, robust schemes delivering quantifiable, secure and cost‐effective benefits (Bonn et al., 2014). The main challenges to private sector finance inflows will be issues of cost‐effectiveness, permanence, leakage, additionality, handling of co‐benefits and the fact that many schemes focus on enhanced removals rather than avoided emissions. Viability will depend strongly upon the C commodity price (Ferré et al., 2019). C sequestration achieved through land‐use change will always be open to future reversals, so payments may need to focus on avoided emissions as opposed to removals (Evans et al., 2020). Displacement of food production will also carry risks of displacing environmental impacts elsewhere (Evans et al., 2020). Legislatively compelled rewetting would not be suitable for private finance schemes and care will be needed to ensure that co‐benefits (e.g. water quality or flood regulation) are adequately accounted for, through either service bundling or payment layering, so that land managers receive fair remuneration (Bonn et al., 2014). Development of regional C markets such as MoorFutures^®^ in Germany and the Peatland Code in the United Kingdom show promise (Bonn et al., 2014). However, work is still needed to strengthen the evidence base (Evans et al., 2014) and ensure sufficient regulatory support (Bonn et al., 2014).

**FIGURE 5:**
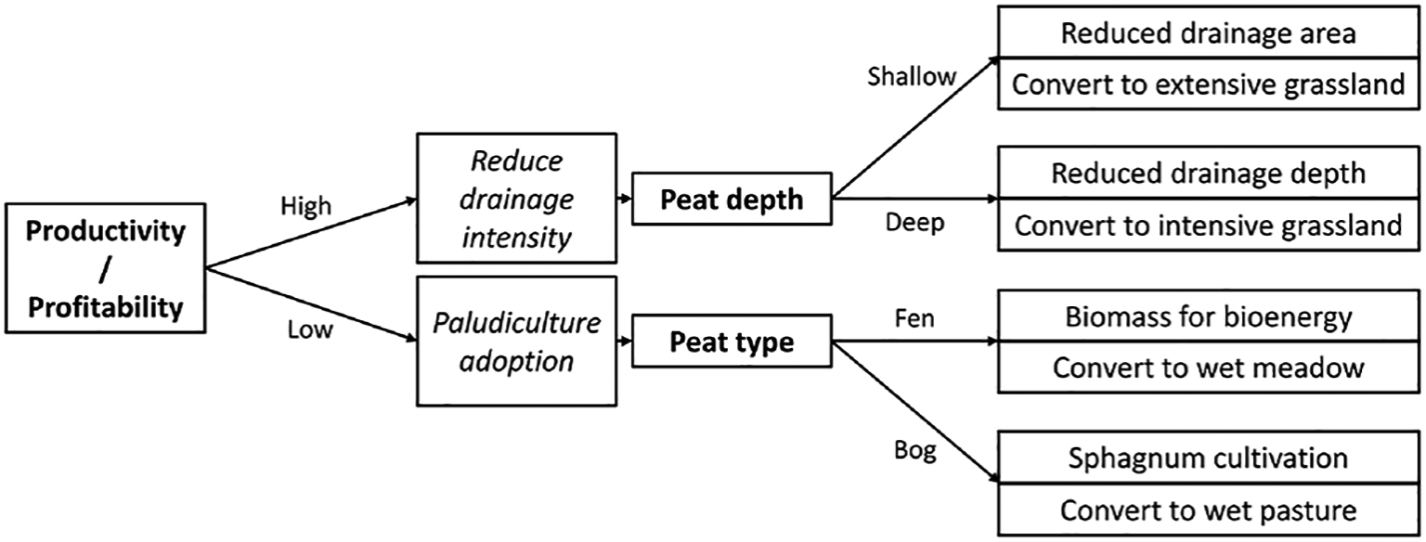
Example decision‐making tree for wetland agriculture adoption. This is based on the approach of Kekkonen et al. (2019), who demonstrated that accurate spatial data combined with an appropriate decision‐making tree could provide a practical tool for land‐use planning. In practice, the decision criteria selected and boundaries between classifications (e.g. deep/shallow peat) would need to be defined appropriately for the physical and socio‐economic conditions of the nation/region in question. Those chosen here, along with the options presented in the right hand column of boxes do not represent an exhaustive list and are presented for illustrative purposes only. Bold text indicates decision criteria. Italics indicate wetland agriculture sub‐categories. Peatland restoration (not shown) would also be an essential component of wider responsible peatland management strategies

Governments may have a role to play in scenarios that are not cost‐effective for private C markets (due to high implementation costs/limited benefits). They may also be required to regulate private schemes and provide longer‐term security to land managers who will be bearing outsized personal risks to produce public goods (Buschmann et al., 2020; Reed et al., 2020). There will likely be a need for some pro‐environmental regulation (e.g. on maximum baseline WTDs) to ensure adequate improvements in environmental outcomes. However, there is a balance to be struck, as excessive regulation or government financial support will impede private sector funding (Bonn et al., 2014). Regulation could be balanced by incentivising/support mechanisms; recognising that much drainage occurred before the environmental consequences were understood and supporting land managers to be active participants in the solution. Future emissions from drained peatlands could be seen as the responsibility of land managers who continue drainage‐based management and the cost of these emissions could be recovered under the ‘polluter pays principle’. This would be politically polarising but would fundamentally shift the economic landscape. A UK analysis balancing agricultural income against the cost of GHG emissions (and payments for net sequestration) indicated that peatland restoration and extensive grassland adoption could offer net benefits of ~£650 GBP ha ^−1^ yr^−1^ on average over continued intensive arable production (2012 prices; Graves & Morris, 2013). These values would likely be lower on the most productive sites but could be enhanced by inclusion of payments for other ecosystem services and would favour wetland agriculture adoption and peatland restoration. Additionally, reduction or cessation of subsidy for drainage‐based agriculture may incentivise adoption of pro‐environmental alternatives (Ziegler et al., 2021). Decisions on how the costs of peatland GHG emissions are treated (penalties vs. payments for avoided emissions) and allocated (to land managers, governments and/or private investors) will be a major driver of eventual outcomes. Land‐use planning will require clear, robust decision‐making frameworks (Figure 5), accurate spatial data and flexible responses to regional differences (Kekkonen et al., 2019). Developing a coherent policy framework (aligning goals between different policy areas and levels of governance) and ensuring the absence of legislative obstacles will be key to efficient implementation (Buschmann et al., 2020; Regina et al., 2016).

The socio‐economic analysis presented draws strongly from European sources due to a geographical publication bias, which in turn reflects the relatively widespread and longer‐term drainage and cultivation of European peatlands compared with most other mid‐latitude regions. The relative profitability of agricultural peatlands, availability of subsidies/private funding flows and viability of different regulatory strategies will vary substantially across mid‐latitude regions. Local (national or regional) solutions will need to be found that suit the circumstances, character and values of each region. Land managers will ultimately be responsible for implementing wetland agriculture strategies. However, it is clear that researchers, policymakers and private investors will all need to play key roles if wetland agriculture adoption is to be sufficiently successful and widespread to deliver a meaningful contribution to meeting climate goals. Given the complexity and urgency of the challenge, and the need to navigate risks and uncertainties, there is a real need for compromise, collaboration and cooperation between stakeholder groups, to ensure positive outcomes.

## 5 CONCLUSIONS

Application of conventional, drainage‐based agricultural practices to mid‐latitude lowland peatlands has produced highly productive agroecosystems. However, these systems are losing irrecoverable C and represent a disproportionately large source of GHG emissions relative to the area they occupy. There is widespread agreement that substantial reductions in GHG emissions will be required to mitigate potential harms stemming from global climate change. Emission reduction pressures will inevitably be focused on hotspot sectors and consequently, both international and national climate policies are driving efforts to balance productivity with improved environmental outcomes for agricultural lowland peatlands. Peatlands are naturally wet systems and as a result, hydrology is fundamental to their function and management. Maximising progress towards GHG emission targets will require management for peatlands with near‐surface WTDs, suppressing rates of SOM mineralisation and CO_2_ emissions, which account for the bulk of GHG emissions from agricultural peatlands. Socio‐economic constraints mean that full rewetting/restoration will not always be immediately possible. Paludiculture appears to be a highly effective strategy for GHG mitigation but it is unclear at what scale it can be economically viable in the current economic/regulatory landscape. Consequently, development of reduced drainage intensity systems, involving compromise between food production and climate mitigation, will also be important to help minimise climate impacts until societal/economic conditions favour strategies that can completely halt emissions. Smaller reductions in agricultural profitability (relative to completely rewetted systems) will be needed in recognition of the constraints faced by land managers in the current economic landscape, to facilitate the delivery of admittedly more limited reductions in rates of GHG emissions per unit area over the large areas required to produce meaningful change.

Wetland agriculture adoption will require a range of agronomic, hydrological and socio‐economic challenges to be overcome and inevitably presents risks to land managers. The viability of the different options identified is not yet clear. Therefore, assessments of viability and adoption risks represent important priorities for research, which must focus on the creation of workable solutions and avoid over simplistic idealism in the face of complex realities. The need for change creates uncertainty, which is exacerbated by the urgency of the response required by climate change and creates a challenging environment for land managers and policymakers alike. However, it is easy to focus on the risks and dismiss the opportunities. The development and implementation of well‐designed wetland agriculture strategies could present an opportunity to create more resilient production systems, improve delivery of wider environmental benefits and protect valuable peatland ecosystems for everyone's future benefit. Combining wetland agriculture systems with restored semi‐natural sites, constructed wetlands (e.g. for water treatment) and renewable energy systems (e.g. solar/wind), could allow us to create thriving, innovative and green wetland landscapes; delivering a vital contribution to global climate change mitigation efforts.

## Supporting information

Supplementary Material

Supplementary Material

Supplementary Material

Supplementary Material

## Data Availability

Data sharing is not applicable to this article as no new data were created or analyzed in this study.
