# Supplementary material for: Responsible agriculture must adapt to the wetland character of mid‐latitude peatlands
Source: Glob Chang Biol. 2022 Mar 17;28(12):3795–811. doi: 10.1111/gcb.16152 (PMC9314663; doi:10.1111/gcb.16152)
Supplement: Supplementary file 1 — Supplementary Material [file GCB-28-3795-s001.docx]

**S1. Mid-latitude peatlands definition**

We have broadly defined mid-latitude peatlands as non-tropical and non-polar for the purposes of this analysis. The ‘tropical’ boundary can be represented conveniently by the tropics of Cancer (23.5° N) and Capricorn (23.5° S), due to relatively low peatland coverage at these specific latitudes (Fig. S1.1). The ‘polar’ boundary is less easily defined in terms of latitude, due to the large peatland area seen at higher latitudes in the Northern Hemisphere. Therefore, we did not use the Arctic and Antarctic circles (66.5° N and S respectively) to guide our ‘polar’ boundaries. Given the importance of climatic conditions in modulating peatland function, a better indicator of the ‘polar’ boundaries is given by the limits of the polar climate zones (ET and EF in Fig. S1.2; Peel et al., 2007). This definition includes large areas of boreal peatland in the northern hemisphere particularly, where climatic conditions are not currently viable for agricultural use. However, given the potential for expansion of the global agricultural area towards the poles by 1200 km by the year 2099 (an area of 5 million km^2^), these peatlands could be highly vulnerable to future drainage (Unc et al., 2021). Their inclusion thus represents an acknowledgement that climate zones and viable activities are not fixed over time. It also represents – due to the vast carbon stocks in these regions – a clear reason why a policy of ‘no further peatland drainage’ is essential if overall peatland greenhouse gas emissions are to be halted. An argument can be made for defining the ‘tropical’ boundary using climate zones also. The main boundary case of note would be the Florida Everglades (USA). The inclusion of this location in our definition is likely constructive as their socio-economic circumstances are more closely aligned with other mid-latitude peatlands in the USA. However, due to climatic conditions, soil organic matter mineralisation and soil respiration rates may be higher than at most other mid-latitude sites and it may represent something of an outlier. It is important to note that our analysis was not heavily reliant on data from this location and so this boundary decision does not affect our conclusions.


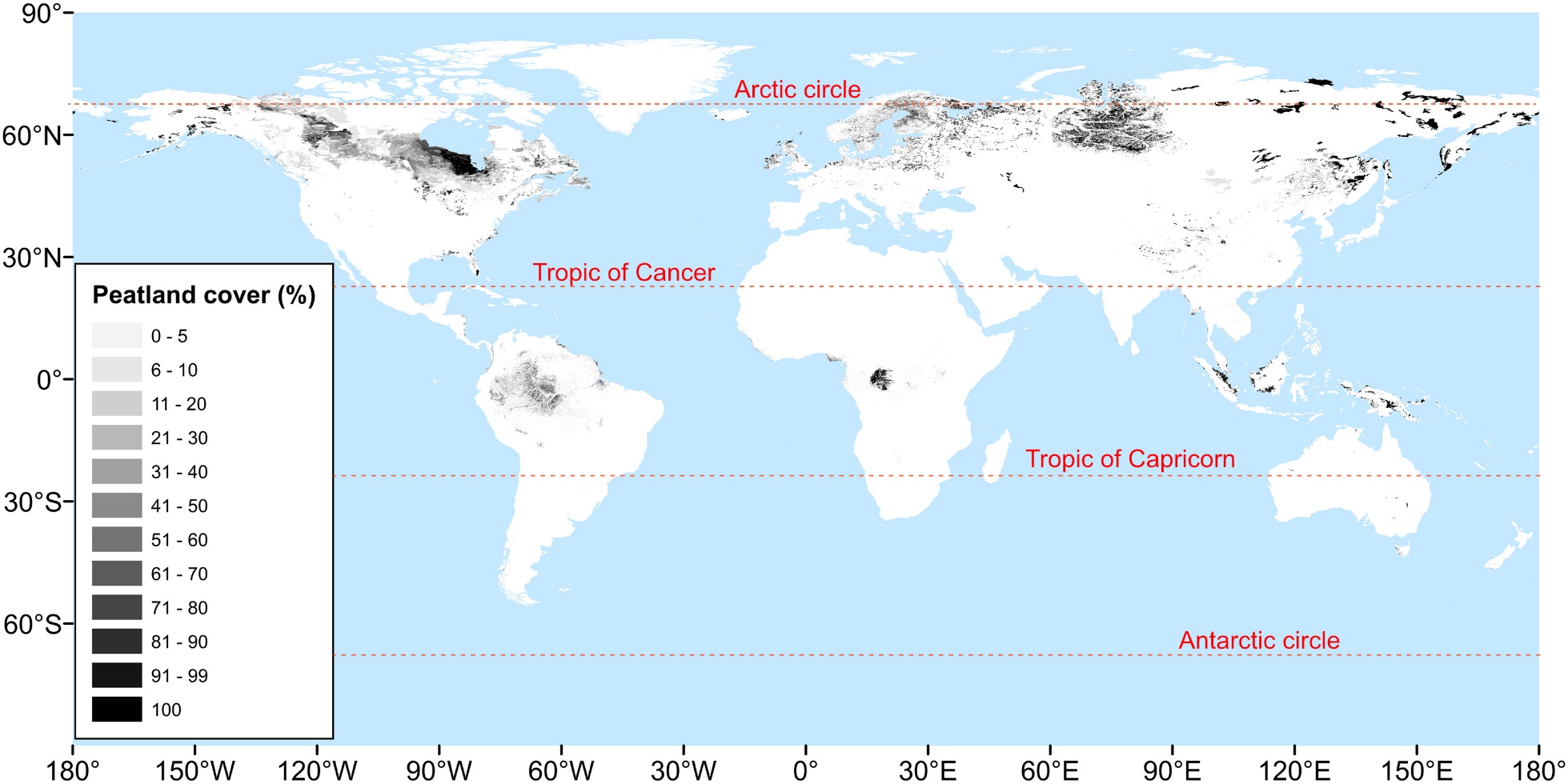


**Figure S1.1. Global peatland distribution derived from PEATMAP.** The Arctic and Antarctic circles (66.5° North and South respectively) along with the Tropics of Cancer and Capricorn (23.5° North and South respectively) are hand drawn additions to the original figure, shown as red dashed lines for the readers convenience (the locations are not exact but indicative). Reprinted with permission from Xu et al. (2018), © 2018 Elsevier.


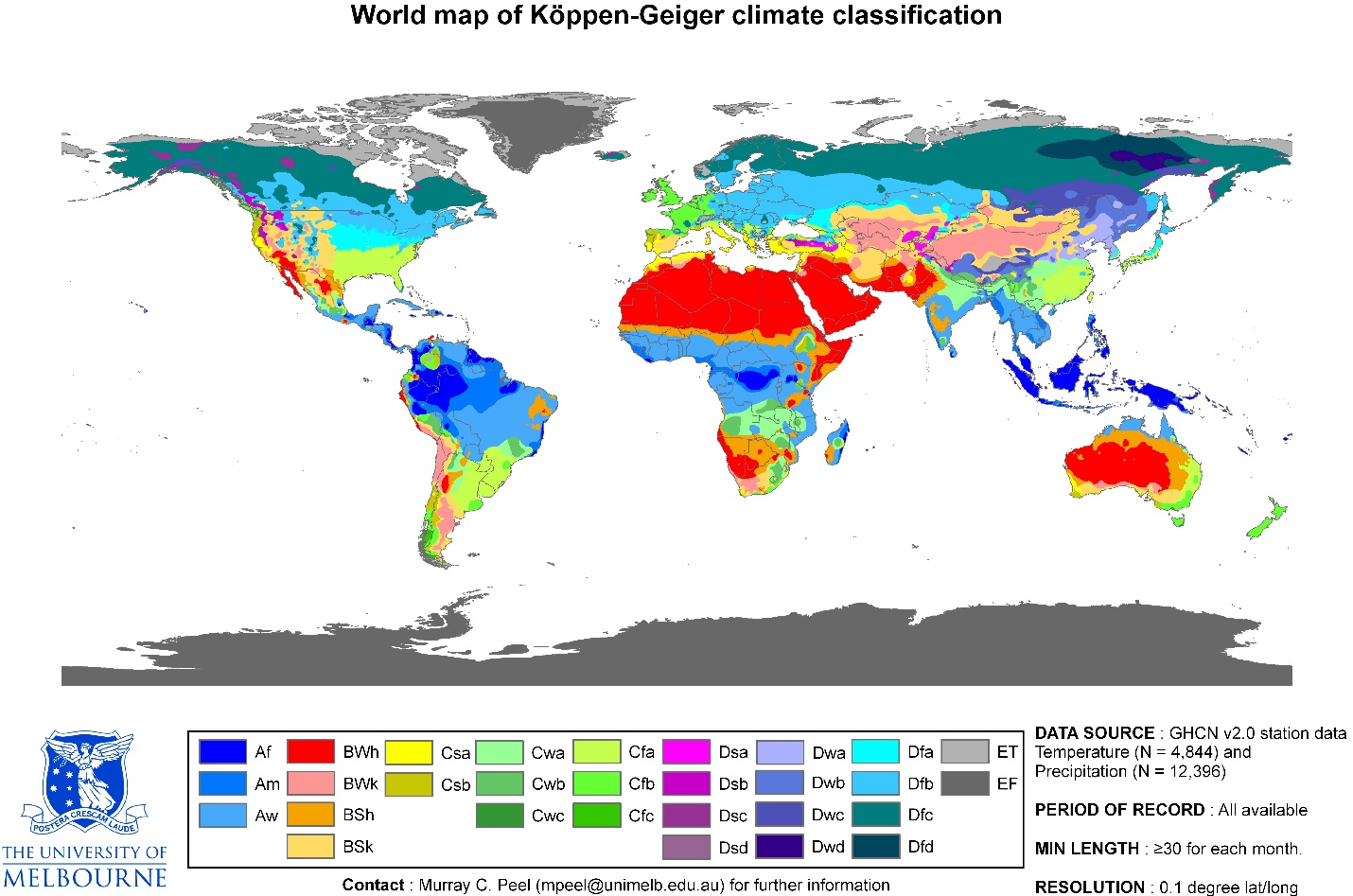


**Figure S1.2. World map of the Köppen-Geiger climate classification.** The first letters in each category signify: A = Tropical, B = Arid, C = Temperate, D = Cold (Boreal), E = Polar. Further letters indicate temperature/rainfall/habitat descriptors and are described fully in the original publication. The area shown as Dfc best approximates those regions that whilst not currently climatically favourable for agriculture, would see increasing development with climate warming. Image reprinted from Peel et al. (2007), © Peel, M.C., Finlayson, B.L., McMahon, T.A., 2007. This work is licensed under the [Creative Commons Attribution-NonCommercial-ShareAlike 2.5 License](https://eur01.safelinks.protection.outlook.com/?url=https%3A%2F%2Fcreativecommons.org%2Flicenses%2Fby-nc-sa%2F2.5%2F&data=04%7C01%7Cb.freeman%40bangor.ac.uk%7Ca4c4aaffefe74e652aa908d9fae6cca9%7Cc6474c55a9234d2a9bd4ece37148dbb2%7C0%7C1%7C637816691456809789%7CUnknown%7CTWFpbGZsb3d8eyJWIjoiMC4wLjAwMDAiLCJQIjoiV2luMzIiLCJBTiI6Ik1haWwiLCJXVCI6Mn0%3D%7C0&sdata=q36QbqRZ9jq5DqmU5v8cSd01lMBFjuOclsJJeMSPiZk%3D&reserved=0). Original image downloadable at: <https://hess.copernicus.org/articles/11/1633/2007/hess-11-1633-2007-supplement.zip>

**Additional references**

Peel, M.C., Finlayson, B.L., McMahon, T.A., 2007. Updated world map of the Köppen-Geiger climate classification. Hydrology and Earth System Sciences, 11, 1633-1644. <https://doi.org/10.5194/hess-11-1633-2007>

Unc, A., Altdorff, D., Abakumov, E., Adl, S., Baldursson, S., Bechtold, M., Cattani, D.J., Firbank, L.G., Grand, S., Guðjónsdóttir, M., Kallenbach, C., Kedir, A.J., Li, P., McKenzie, D.B., Msira, D., Nagano, H., Neher, D.A., Niemi, J., Oelbermann, M., Overgård Lehmann, J., Parsons, D., Quideau, S., Sharkhuu, A., Smreczak, B., Sorvali, J., Vallotton, J.D., Whalen, J.K., Young, E.H., Zhang, M., Borchard, N., 2021. Expansion of Agriculture in Northern Cold-Climate Regions: A Cross-Sectoral Perspective on Opportunities and Challenges. Frontiers in Sustainable Food Systems, 5, 663448. <https://doi.org/10.3389/fsufs.2021.663448>

Xu, J., Morris, P.J., Liu, J., Holden, J., 2018. PEATMAP: Refining estimates of global peatland distribution based on a meta-analysis. CATENA, 160, 134-140. <https://doi.org/10.1016/j.catena.2017.09.010>
