# Supplementary material for: Responsible agriculture must adapt to the wetland character of mid‐latitude peatlands
Source: Glob Chang Biol. 2022 Mar 17;28(12):3795–811. doi: 10.1111/gcb.16152 (PMC9314663; doi:10.1111/gcb.16152)
Supplement: Supplementary file 2 — Supplementary Material [file GCB-28-3795-s002.docx]

**S2. Subsidence analysis**

Subsidence values from a number of studies are collated in Table S2.1. The median subsidence rate observed is 2.0 cm yr^-1^ (Quartiles = 1.3 – 2.7, n = 48). Median subsidence rates observed under cropland were slightly higher (2.1 cm yr^-1^; Quartiles = 1.5 – 3.0, n = 21) than for grassland (1.0 cm yr^-1^; Quartiles = 0.7 – 1.5, n = 13). Any difference would likely be a reflection of differences in drainage intensity between land uses, as water table depths appear to be the dominant factor determining subsidence rates (Evans et al., 2019). A higher median subsidence rate was observed in North American studies (2.5 cm yr^-1^; Quartiles = 1.9 – 3.1, n = 19) than European studies (1.4 cm yr^-1^; Quartiles = 0.8 – 2.1; n = 25). This result is almost certainly biased by the inclusion of warmer climate peatlands (e.g. Florida Everglades and Sacramento-San Joaquin Delta) in the North American sample, where mineralisation rates would be greater due to enhanced reaction kinetics (Stephens and Stewart, 1969). However, it serves as a useful demonstration of the potential for future climate warming to increase environmental impacts resulting from drainage and highlights the universal fragility of peatlands.

**Table S2.1. Annual subsidence values for mid-latitude peatlands drained for agriculture.** This table is an update of previous research published in Evans et al. (2019).

| **Region** | **Location** | **Land use** | **Subsidence (cm yr^-1^)** | **Source** |
| --- | --- | --- | --- | --- |
| **Asia** | Japan | Arable | 3.00 | Miyaji et al. (1995) |
| **Australasia** | New Zealand | Grassland | 3.40 | Schipper and McLeod (2002) |
|  | New Zealand | Grassland | 2.56 | Fitzgerald and McLeod (2004) |
|  | New Zealand | Grassland | 1.90 | Pronger et al. (2014) |
| **Europe** | Belarus | Unknown | 2.10 | Armentano (1979) |
|  | Germany | Arable | 2.70 | Eggelsmann and Bartels (1975) |
|  | Germany | Grassland | 0.67 | Eggelsmann and Bartels (1975) |
|  | Germany | Grassland | 0.50 | Eggelsmann (1976) |
|  | Germany | Grassland | 0.83 | Kluge et al, (2008) |
|  | Ireland | Unknown | 1.80 | Armentano (1979) |
|  | Italy | Arable | 1.75 | Gambolati et al. (2005) |
|  | Italy | Arable | 0.75 | Zanello et al. (2011) |
|  | Netherlands | Grassland | 0.88 | Schothorst (1977) |
|  | Netherlands | Unknown | 0.70 | Armentano (1979) |
|  | Netherlands | Unknown | 1.35 | Armentano (1979) |
|  | Norway | Grassland | 2.50 | Grønlund et al. (2008) |
|  | Norway | Unknown | 2.50 | Armentano (1979) |
|  | Poland | Grassland | 0.60 | Grzywna (2017) |
|  | Sweden | Arable | 2.50 | Berglund and Berglund (2010) |
|  | Sweden | Arable | 1.50 | Berglund and Berglund (2010) |
|  | Sweden | Grassland | 1.00 | Berglund and Berglund (2010) |
|  | Switzerland | Arable | 1.26 | Leifeld et al. (2011) |
|  | Switzerland | Unknown | 1.27 | Wüst-Galley et al. (2019) |
|  | Ukraine | Grassland | 2.00 | Lipka et al. (2017) |
|  | UK | Arable | 1.37 | Richardson and Smith (1977) |
|  | UK | Arable | 3.05 | Hutchinson (1980) |
|  | UK | Arable | 1.48 | Dawson et al. (2010) |
|  | UK | Grassland | 0.62 | Brunning (2001) |
|  | UK | Unknown | 2.75 | Armentano (1979) |
| **North America** | Canada (Ontario) | Arable | 3.30 | Mirza and Irwin (1964) |
|  | Canada (Quebec) | Arable | 2.07 | Millette (1976) |
|  | Canada (Quebec) | Arable | 2.50 | Mathur et al. (1982) |
|  | USA (CA) | Arable | 0.83 | Deverel et al. (2016) |
|  | USA (CA) | Grassland | 2.20 | Deverel and Leighton (2010) |
|  | USA (CA) | Unknown | 5.35 | Armentano (1979) |
|  | USA (FL) | Arable | 3.18 | Stephens (1956) |
|  | USA (FL) | Arable | 3.00 | Stephens et al. (1984) |
|  | USA (FL) | Arable | 1.45 | Shih et al. (1998) |
|  | USA (FL) | Arable | 1.40 | Wright and Snyder (2009) |
|  | USA (FL) | Arable | 1.82 | Aich et al. (2013) |
|  | USA (FL) | Unknown | 2.70 | Armentano (1979) |
|  | USA (FL) | Unknown | 3.45 | Armentano (1979) |
|  | USA (IN) | Arable | 2.26 | Jongedyk et al. (1950) |
|  | USA (IN) | Unknown | 1.85 | Armentano (1979) |
|  | USA (LA) | Unknown | 3.00 | Armentano (1979) |
|  | USA (MI) | Unknown | 1.85 | Armentano (1979) |
|  | USA (NC) | Arable | 4.00 | Ewing and Vepraskas (2006) |
|  | USA (NY) | Unknown | 2.50 | Armentano (1979) |

**Additional references**

Aich, S., McVoy, C.W., Dreschel, T.W., Santamaria, F., 2013. Estimating soil subsidence and carbon loss in the Everglades Agricultural Area, Florida using geospatial techniques. Agriculture, Ecosystems and Environment, 171, 124-133. <https://doi.org/10.1016/j.agee.2013.03.017>

Armentano, T.V., 1979. Role of organic soils in the world carbon cycle: problem definition and research needs. Technical Report DOE/EV/10040-2, The Institute of Ecology, Indianapolis, Indiana, United States. <https://doi.org/10.2172/5337473>

Berglund, Ö., Berglund, K., 2010. Distribution and cultivation intensity of agricultural peat and gyttja soils in Sweden and estimation of greenhouse gas emissions from cultivated peat soils. Geoderma, 154, 173–180. <https://doi.org/10.1016/j.geoderma.2008.11.035>

Brunning, R., 2001. Archaeology and peat wastage on the Somerset Moors. Report to the Environment Agency, Somerset County Council, Taunton.

Dawson, Q., Kechavarzi, C., Leeds-Harrison, P.B., Burton, R.G.O., 2010. Subsidence and degradation of agricultural peatlands in the Fenlands of Norfolk, UK. Geoderma, 154, 181–187. <https://doi.org/10.1016/j.geoderma.2009.09.017>

Deverel, S.J., Leighton, D.A., 2010. Historic, recent, and future subsidence, Sacramento- San Joaquin Delta, California, USA. San Francisco Estuary Watershed Science, 8, 1–23. <https://doi.org/10.15447/sfews.2010v8iss2art1>

Deverel, S. J., Ingrum, T., Leighton, D., 2016. Present-day oxidative subsidence of organic soils and mitigation in the Sacramento-San Joaquin Delta, California, USA. Hydrogeology Journal, 24, 569–586. <https://doi.org/10.1007/s10040-016-1391-1>

Eggelsmann, R., Bartels, R., 1975. Oxidativer torfverzehr im niedermoor in abhängigkeit von entwässerung, nutzung und dünung. Mitteilungen der Deutsche Bodenkundliche Gessellschaft. 22, 215–221.

Eggelsman, R., 1976. Peat consumption under influence of climate, soil condition and utilization. Proceedings of the Fifth International Peat Congress, Poznań, Poland, 1, 233-247.

Ewing, J.M., Vepraskas, M.J., 2006. Estimating primary and secondary subsidence in an organic soil 15, 20, and 30 years after drainage. Wetlands, 26, 119–130. [https://doi.org/10.1672/0277-5212(2006)26[119:epassi]2.0.co;2](https://doi.org/10.1672/0277-5212(2006)26%5b119:epassi%5d2.0.co;2)

Fitzgerald, N., McLeod, M., 2004. Subsidence rates of peat since 1924 in the Rukuhia Swamp, in: Technical report 2004/20. Environment Waikato, Waikato, New Zealand.

Gambolati, G., Putti, M., Teatini, P., Camporese, M., Ferraris, S., Gasparetto Stori, G., Nicoletti, V., Silvestri, S., Rizzetto, F., Tosi, L., 2005. Peat land oxidation enhances subsidence in the Venice watershed. Eos, 86, 217-220. <https://doi.org/10.1029/2005EO230001>

Grønlund, A., Hauge, A., Hovde, A., Rasse, D.P., 2008. Carbon loss estimates from cultivated peat soils in Norway: A comparison of three methods. Nutrient Cycling in Agroecosystems, 81, 157–167. <https://doi.org/10.1007/s10705-008-9171-5>

Grzywna, A., 2017. The degree of peatland subsidence resulting from drainage of land. Environmental Earth Sciences, 76, 1–8. <https://doi.org/10.1007/s12665-017-6869-1>

Jongedyk, H.A., Hickock, R.B., Mayer, I.D., Ellis, N.K., 1950. Subsidence of muck soils in northern Indiana. Purdue University Agriculture Experimental Station Special Circular, pp. 366.

Kluge, B., Wessolek, G., Facklam, M., Lorenz, M., Schwärzel, K., 2008. Long-term carbon loss and CO2-C release of drained peatland soils in northeast Germany. European Journal of Soil Science, 59, 1076-1086. <https://doi.org/10.1111/j.1365-2389.2008.01079.x>

Lipka, K., Zając, E., Hlotov, V., Siejka, Z., 2017. Disappearance rate of a peatland in Dublany near Lviv (Ukraine) drained in 19th century. Mires and Peat, 19, 1–15. <https://doi.org/10.19189/MaP.2017.OMB.279>

Mathur, S.P., Levesque, M.P., Richard, P.J.H., 1982. The establishment of synchrony between subsurface layers and estimation of overall subsidence of cultivated organic soils by a palynological method. Canadian Journal of Soil Science, 62, 427–431. <https://doi.org/10.4141/cjss82-047>

Millette, J.A. (2010). Subsidence of an organic soil in southwestern Quebec. Canadian Journal of Soil Science, 56, 499–500. <https://doi.org/10.4141/cjss76-058>

Mirza, C., Irwin, R.W., 1964. Determination of subsidence of an organic soil in southern Ontario. Canadian Journal of Soil Science, 44, 248–253. <https://doi.org/10.4141/cjss64-035>

Miyaji, N., Kohyama, K., Otsuka, H., 1995. Evaluation of peatland in northern Japan in terms of land subsidence. Japan Agricultural Research Quarterly, 29, 95–102.

Pronger, J., Schipper, L.A., Hill, R.B., Campbell, D.I., Mcleod, M., 2014. Subsidence Rates of Drained Agricultural Peatlands in New Zealand and the Relationship with Time since Drainage. Journal of Environmental Quality, 43, 1442-1449. <https://doi.org/10.2134/jeq2013.12.0505>

Richardson, S.J., Smith, J., 1977. Peat Wastage in the East Anglian Fens. Journal of Soil Science, 28, 485–489. <https://doi.org/10.1111/j.1365-2389.1977.tb02256.x>

Schipper, L.A., McLeod, M., 2002. Subsidence rates and carbon loss in peat soils following conversion to pasture in the Waikato Region, New Zealand. Soil Use and Management. 18, 91–93. <https://doi.org/10.1111/j.1475-2743.2002.tb00225.x>

Schothorst, C.J., 1977. Subsidence of low moor peat soils in the western Netherlands. Geoderma, 17, 265-291. <https://doi.org/10.1016/0016-7061(77)90089-1>

Shih, S.F., Glaz, B., Barnes, R.E., 1998. Subsidence of organic soils in the Everglades agricultural area during the past 19 years. Soil and Crop Science Society of Florida Proceedings, 57, 20–29.

Stephens, J.C., 1956. Subsidence of Organic Soils in the Florida Everglades. Soil Science Society of America Journal, 20, 77–80. <https://doi.org/10.2136/sssaj1956.03615995002000010019x>

Stephens, J.C., Allen, L.H., Chen, E., 1984. Organic Soil Subsidence, in: Holzer, T.L. (Ed.), Man-Induced Land Subsidence. Geological Society of America, Boulder, Colorado, USA. <https://doi.org/10.1130/REG6-p107>

Wüst-Galley, C., Grünig, A., Leifeld, J., 2019. Land use-driven historical soil carbon losses in Swiss peatlands. Landscape Ecology. <https://doi.org/10.1007/s10980-019-00941-5>

Wright, A.L., Snyder, G.H., 2009. Soil subsidence in the Everglades agricultural area. Soil and Water Department University of Florida/IFAS Extension*,* SL 311, 1–3.

Zanello, F., Teatini, P., Putti, M., Gambolati, G., 2011. Long term peatland subsidence: Experimental study and modeling scenarios in the Venice coastland. Journal of Geophysical Research: Earth Surface, 116, F04002. <https://doi.org/10.1029/2011JF002010>
