# Supplementary material for: Responsible agriculture must adapt to the wetland character of mid‐latitude peatlands
Source: Glob Chang Biol. 2022 Mar 17;28(12):3795–811. doi: 10.1111/gcb.16152 (PMC9314663; doi:10.1111/gcb.16152)
Supplement: Supplementary file 3 — Supplementary Material [file GCB-28-3795-s004.docx]

**S3. Terrestrial greenhouse gas balance analysis**

Data for this analysis were drawn from the IPCC Wetland Supplement (Drösler et al., 2014), and two other synthesis studies, estimating Tier 2 emission factors for the UK (Evans et al., 2017) and Germany (Tiemeyer et al., 2020). Total GHG balances were calculated as the sum of terrestrial carbon dioxide (CO_2_), methane (CH_4_) and nitrous oxide (N_2_O) emissions (Table S3.1). We used factors of 3.66412 to convert CO_2_-C to CO_2_ and 1.57112 to convert N_2_O-N to N_2_O. Values for GHGs were converted to CO_2_-e by calculating 100-year global warming potentials using factors of 1, 265 and 28 for CO_2_, N_2_O and CH_4_ respectively as described in IPCC AR5 (Myhre et al., 2013), in line with UN Framework Convention on Climate Change guidelines for national and international emissions reporting (UNFCCC, 2021). Average values for the land used categories were calculated as the arithmetic mean of the available literature values. For the extensive grassland category, we used the values for extensive grassland from Evans et al. (2017) and for shallow-drained, nutrient-rich grassland from the IPCC Wetland Supplement. For the intensive grassland category, we used the values for both deep-drained, nutrient-rich and drained, nutrient-poor grassland from the IPCC Wetland Supplement, along with the value for intensive grassland from Evans et al. (2017) and the value for grassland from Tiemeyer et al. (2020). This analysis focused on temperate and not boreal mid-latitude peatlands as (i) the IPCC tier 1 emission factors do not differentiate between grassland categories for boreal climates and (ii) the tier 2 emission factors available were predominately for temperate regions.

**Table S3.1. Results of terrestrial greenhouse gas balance analysis for temperate agricultural peatlands.** Values for greenhouse gas emissions are reported as t CO_2_-e ha^-1^ yr^-1^ and as a percentage contribution to the total greenhouse gas balance. Sources: 1) Drösler et al. (2014), 2) Evans et al. (2017), 3) Tiemeyer et al. (2020).

| **Land use** | **CO_2_** | **CH_4_** | **N_2_O** | **Total** | **% CO_2_** | **% CH_4_** | **% N_2_O** |
| --- | --- | --- | --- | --- | --- | --- | --- |
| **Cropland^1^** | 29 (24-34) | 0 (-0.1-0.1) | 5.4 (3.4-7.5) | 34.4 | 84.2 | 0 | 15.8 |
| **Cropland^2^** | 26 (15-38) | 0 (0-0.1) | 8.0 (2.8-13.1) | 34.4 | 76.8 | 0.1 | 23.1 |
| **Cropland^3^** | 34 (11-41) | 0.2 (0-0.5) | 4.6 (0.7-16.9) | 38.5 | 87.6 | 0.4 | 12.0 |
| **Grassland, deep-drained, nutrient-rich^1^** | 22 (18-27) | 0.4 (0.1-0.8) | 3.4 (2.0-4.6) | 26.2 | 85.3 | 1.7 | 13.0 |
| **Grassland, drained, nutrient-poor^1^** | 19 (14-25) | 0.1 (0-0.1) | 1.8 (0.8-2.8) | 21.3 | 91.3 | 0.2 | 8.4 |
| **Grassland, shallow-drained, nutrient-rich^1^** | 13 (7-20) | 1.1 (-0.1-2.3) | 0.7 (0.2-1.1) | 14.9 | 88.2 | 7.3 | 4.5 |
| **Grassland intensive^2^** | 23 (14-33) | 0.4 (-0.4-1.2) | 2.5 (1.2-3.8) | 26.4 | 88.9 | 1.6 | 9.5 |
| **Grassland extensive^2^** | 13 (8-19) | 2.0 (0.5-3.6) | 1.3 (0.1-2.6) | 16.6 | 79.6 | 12.3 | 8.0 |
| **Grassland^3^** | 30 (5-40) | 0.3 (0-2.4) | 1.9 (0.1-9.2) | 32.6 | 93.2 | 1.0 | 5.9 |
| **Cropland (average)** | 30 | 0.1 | 6.0 | 35.7 | 82.9 | 0.2 | 17.0 |
| **Grassland intensive (average)** | 24 | 0.3 | 2.4 | 26.6 | 89.7 | 1.1 | 9.2 |
| **Grassland extensive (average)** | 13 | 1.6 | 1.0 | 15.8 | 83.9 | 9.8 | 6.2 |

**Additional references**

UNFCCC, 2021. Common metrics. <https://unfccc.int/process-and-meetings/transparency-and-reporting/methods-for-climate-change-transparency/common-metrics> (accessed: 01/06/21).
