# Supplementary material for: Responsible agriculture must adapt to the wetland character of mid‐latitude peatlands
Source: Glob Chang Biol. 2022 Mar 17;28(12):3795–811. doi: 10.1111/gcb.16152 (PMC9314663; doi:10.1111/gcb.16152)
Supplement: Supplementary file 4 — Supplementary Material [file GCB-28-3795-s003.docx]

**S4. Exponential methane function for Couwenberg et al. (2011) data**

We generated an exponential function for the relationship between methane (CH_4_) and water table depth (WTD) for Couwenberg et al. (2011) in order to estimate the WTD associated with minimising carbon-derived greenhouse gas balances for this study. We digitised data with WTD > 0 m from Figure 2 of Couwenberg et al. (2011) using WebPlotDigitzer 4.5 (Rohatgi, 2010). This produced a dataset with n = 68 (Table S4.1). The original dataset is described as n = 99, with 3 points at WTDs > 0 m. We therefore obtained 71% of the original dataset. Data that we missed will likely have been less influential data on average, because outliers were easily digitised whereas data in well-represented (visually ‘crowded’) regions of the plot were more challenging to extract. Therefore, this dataset provides a reasonable representation of the main features of the original dataset. We fit an exponential function using the *nls* function in R v4.0.4 (R Core Team, 2021). There was clear heterogeneity of variance and non-normality of residuals resulting from greater spread of values at near surface WTDs than at deeper WTDs (see Fig. S4.1). This is likely because vegetation mediates the relationship between WTD and CH_4_ at near-surface WTDs. The original linear relationship from Couwenberg et al. (2011) used only sites with aerenchymatous shunt species and they did not include open vegetation without shunt species or sites with trees in their regression. We included all available data, without adjusting for vegetation because that information was not available. Visual inspection suggests the mean model prediction offers an acceptable description of the trend. It also agrees well with the Tiemeyer et al. (2020) model for rewetted sites, where variation was also clearly much higher at near-surface WTDs. As such, it was deemed adequate for our purpose. Investigation of the role of vegetation in mediating this relationship represents a potentially important future research target. The resulting function was:

$${CH}_{4}=-1.18+203.54e^{-7.74\cdot WTD}$$

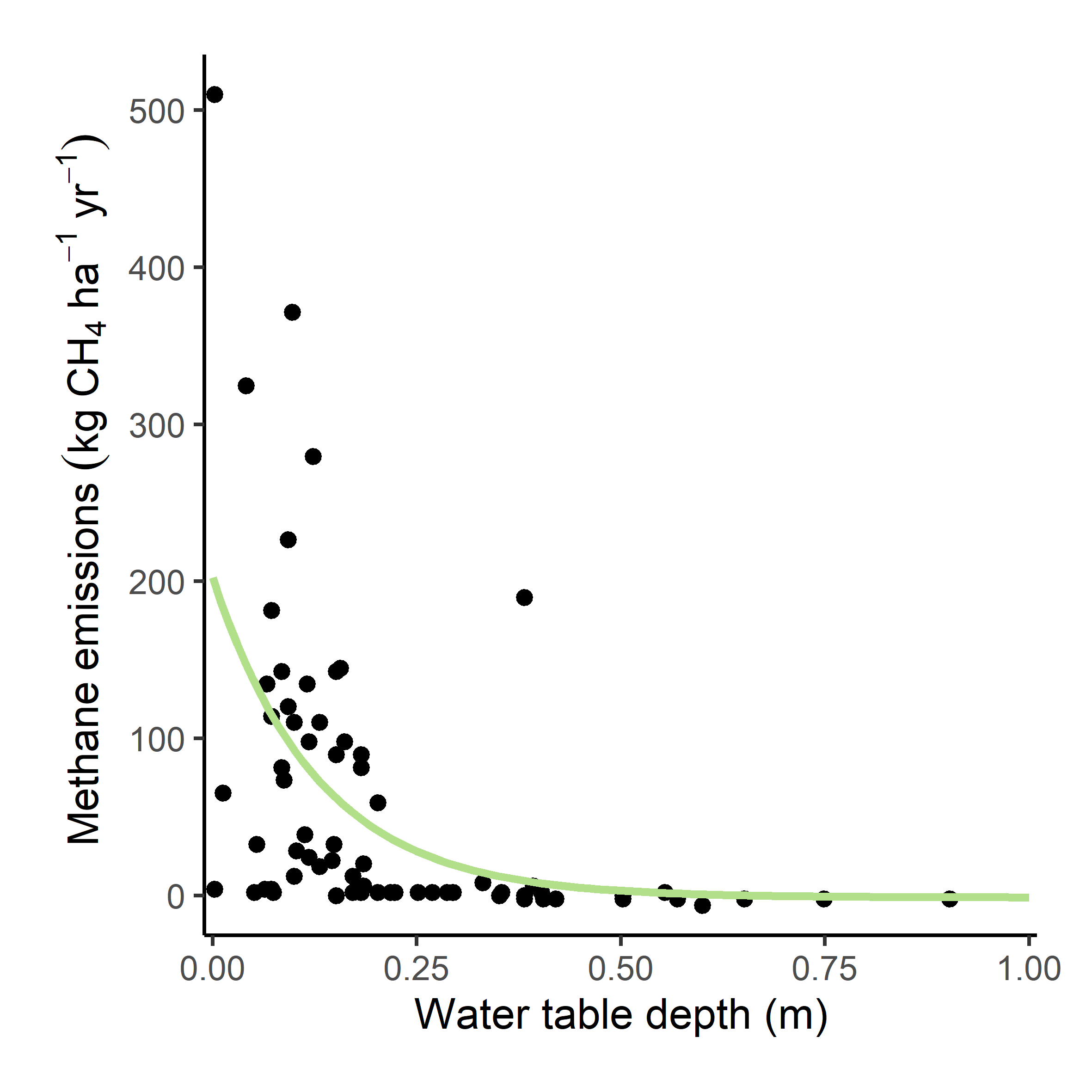


**Figure S4.1. Exponential relationship between methane and water table depth estimated from a subset of Couwenberg et al. (2011) data.**

**Table S4.1. Digitised dataset.**

| **Methane emissions  (kg CH_4_ ha^-1^ yr^-1^)** | **WTD  (m)** |
| --- | --- |
| -2.04082 | 0.902564 |
| -2.04082 | 0.748718 |
| -2.04082 | 0.651282 |
| -6.12245 | 0.6 |
| -2.04082 | 0.569231 |
| 2.040816 | 0.553846 |
| 0 | 0.502564 |
| -2.04082 | 0.502564 |
| -2.04082 | 0.420513 |
| -2.04082 | 0.405128 |
| 2.040816 | 0.402564 |
| 6.122449 | 0.392308 |
| 189.7959 | 0.382051 |
| -2.04082 | 0.382051 |
| 0 | 0.382051 |
| 2.040816 | 0.353846 |
| 0 | 0.351282 |
| 8.163265 | 0.330769 |
| 2.040816 | 0.294872 |
| 2.040816 | 0.287179 |
| 2.040816 | 0.269231 |
| 2.040816 | 0.251282 |
| 2.040816 | 0.223077 |
| 2.040816 | 0.217949 |
| 59.18367 | 0.202564 |
| 2.040816 | 0.202564 |
| 20.40816 | 0.184615 |
| 6.122449 | 0.184615 |
| 89.79592 | 0.182051 |
| 81.63265 | 0.182051 |
| 2.040816 | 0.182051 |
| 2.040816 | 0.171795 |
| 12.2449 | 0.171795 |
| 97.95918 | 0.161538 |
| 144.898 | 0.15641 |
| 142.8571 | 0.151282 |
| 89.79592 | 0.151282 |
| 0 | 0.151282 |
| 32.65306 | 0.148718 |
| 22.44898 | 0.146154 |
| 110.2041 | 0.130769 |
| 18.36735 | 0.130769 |
| 279.5918 | 0.123077 |
| 97.95918 | 0.117949 |
| 24.4898 | 0.117949 |
| 134.6939 | 0.115385 |
| 38.77551 | 0.112821 |
| 28.57143 | 0.102564 |
| 110.2041 | 0.1 |
| 12.2449 | 0.1 |
| 371.4286 | 0.097436 |
| 226.5306 | 0.092308 |
| 120.4082 | 0.092308 |
| 73.46939 | 0.087179 |
| 142.8571 | 0.084615 |
| 81.63265 | 0.084615 |
| 2.040816 | 0.074359 |
| 181.6327 | 0.071795 |
| 114.2857 | 0.071795 |
| 4.081633 | 0.071795 |
| 134.6939 | 0.066667 |
| 4.081633 | 0.064103 |
| 32.65306 | 0.053846 |
| 2.040816 | 0.051282 |
| 324.4898 | 0.041026 |
| 65.30612 | 0.012821 |
| 510.2041 | 0.002564 |
| 4.081633 | 0.002564 |

**Additional references**

R Core Team, 2021. R: A language and environment for statistical computing. R Foundation for Statistical Computing, Vienna, Austria. <https://www.R-project.org/>.

Rohatgi, A., 2010. WebPlotDigitizer. Version 4.5 <https://apps.automeris.io/wpd/>
